# Effects on biodiversity in semi-natural pastures of giving the grazing animals access to additional nutrient sources: a systematic review

**DOI:** 10.1186/s13750-024-00343-4

**Published:** 2024-08-01

**Authors:** Simon Jakobsson, Ida Envall, Jan Bengtsson, Maj Rundlöf, Matilda Svensson, Charlotte Åberg, Regina Lindborg

**Affiliations:** 1 Independent Researcher, Previously Norwegian Institute for Nature Research, Torgarden, P.O. Box 5685, 7485 Trondheim, Norway; 2 The Swedish Research Council for Environment, Agricultural Sciences and Spatial Planning (Formas), Box 1206, 111 82 Stockholm, Sweden; 3 Department of Ecology, Swedish University of Agricultural Sciences (SLU), Box 7044, 750 07 Uppsala, Sweden; 4 Department of Biology, Lund University, 223 62 Lund, Sweden; 5 Department of Physical Geography, Stockholm University, 106 91 Stockholm, Sweden

**Keywords:** Conservation, Grazing management, Nutrient addition, Plant species richness, Sustainable farming

## Abstract

**Background:**

Traditionally managed semi-natural pastures are recognised for their high biodiversity. One drawback is that these pastures are often low in fodder production and hence rather unprofitable, which may lead to abandonment. Two ways to increase production and profitability and maintain grazing are to (i) offer the grazers supplementary feed, or (ii) co-enclose the semi-natural pasture with an improved pasture. Both practices may transfer nutrients to the semi-natural pasture, with potential negative effects on biodiversity. This systematic review aimed to analyse the available evidence concerning the following primary question: “What is the effect of giving grazers access to additional nutrient sources on biodiversity in semi-natural pastures?” (Q1). We also used two supporting questions: “What is the effect of giving grazers access to additional nutrient sources on nutrient status of the soils of semi-natural pastures?” (Q2) and “How do the grazers of semi-natural pastures behave while having access to additional nutrient sources?” (Q3).

**Methods:**

Searches for peer-reviewed and grey literature were made using bibliographic databases, search engines, specialist websites, and stakeholder contacts. Literature was screened for relevance according to predefined eligibility criteria, and critical appraisal was performed using the tool CEECAT. A database of the relevant studies was compiled. Descriptive information about the evidence base is presented in tables and an interactive evidence atlas. Because of absent study setup replication, Q1 and Q2 were not analysed quantitatively. However, sample size allowed the use of mixed modelling to quantitatively analyse Q3 regarding the effects of (i) co-enclosing an improved pasture on grazers’ electivity for the improved area, and (ii) supplementary feed on the forage intake of grazers.

**Review findings:**

A total of 12 articles on the effects of supplementary feeding and 19 on the effects of co-enclosing an improved pasture were included, of which some targeted multiple review questions. Because of the limited literature, it is not possible to draw any conclusions concerning the effects on biodiversity (Q1) or nutritional status (Q2) in semi-natural pastures. For Q3, 28 studies fulfilled our criteria, of which 18 investigated the behaviour of grazers related to co-enclosing an improved pasture, and 10 investigated their forage intake while having access to supplementary fodder. The results show that all grazer species except goats preferred grazing in the improved areas regardless of whether they were grazing together with other grazer species or not. We found no effect of supplementary feeding on forage intake of the grazers.

**Conclusions:**

We detected a knowledge gap concerning the effects of the two additional nutrient sources on semi-natural pasture biodiversity (Q1) and nutrient status (Q2), which points toward further research needs. Analysis of Q3 showed that grazers prefer to graze improved compared to semi-natural pasture areas. However, how this behaviour subsequently affects nutrient transport and biodiversity is unclear and cannot be translated into management recommendations. To gain better knowledge about the primary question of our review, research focusing specifically on this question is needed. We provide suggestions for how such studies could be designed, including spatio-temporal setup, and key management and environmental conditions to consider.

**Supplementary Information:**

The online version contains supplementary material available at 10.1186/s13750-024-00343-4.

## Background

Intensified agriculture is a major threat to biodiversity, and securing sustainable food production and protecting farmland biodiversity are currently among the most critical global environmental issues in the conservation agenda [1, 2]. This is emphasised by, for example, the European Green Deal and United Nations [3, 4]. Expanding the use of grasslands that cannot be used for crop production, because of low productivity or difficult terrain, has been suggested as one way to merge these goals on food production and biodiversity protection [5]. Extensively managed grasslands have played an important role for millennia as areas producing fodder for animals and promoting biodiversity [6, 7]. Whereas the impact of high-intensity grazing may be negative for biodiversity and ecosystem functioning [8–11], low-intensity grazing often maintains and supports it [12, 13].

Traditional low-input grazing systems, such as semi-natural pastures, are recognised for their high biodiversity across multiple organism groups [14]. In particular, they show a high plant species richness [15]. Due to their high overall biodiversity, but also due to their cultural legacy, semi-natural pastures are seen as part of High Nature Value farmlands within the EU's Common Agricultural Policy, and many of them are listed as Annex I habitats in the Habitat's Directive [16]. One drawback is that these pastures are often low in productivity and hence rather unprofitable. With the shift towards more profitable agricultural practices, more than 90% of the semi-natural pastures have been lost since the 1930s in northern Europe, due to abandonment, land use conversion or intensification [17]. To increase profitability, high-productive improved pastures have replaced previous semi-natural pastures in many agricultural systems [18, 19]. These techno-economically improved pastures are usually much more species-poor.

Two ways to increase profitability of semi-natural pastures without fertilising the pasture vegetation, are to offer the animals supplementary feed, or to fence the semi-natural pasture into the same enclosure as an improved pasture. The latter practice may be economically beneficial also for reasons other than increasing fodder availability, as larger pasture areas may be grazed to a lower fence cost per unit area [20]. However, both these practices have long been considered controversial by authorities and practitioners, due to their potential negative effects on biodiversity [21]. Semi-natural pastures are often nutrient-poor, enabling stress-tolerant plant species to persist. This is one of the reasons why semi-natural pastures are rich in plant species. In fertilised, improved pastures, on the other hand, a few competitive generalist species often outcompete other species [8, 22]. Hence, a general perception is that introducing additional nutrients into the system, either through transfer of nutrients from adjacent improved pastures or from supplementary feed, should be avoided.

**Fig. 1:**
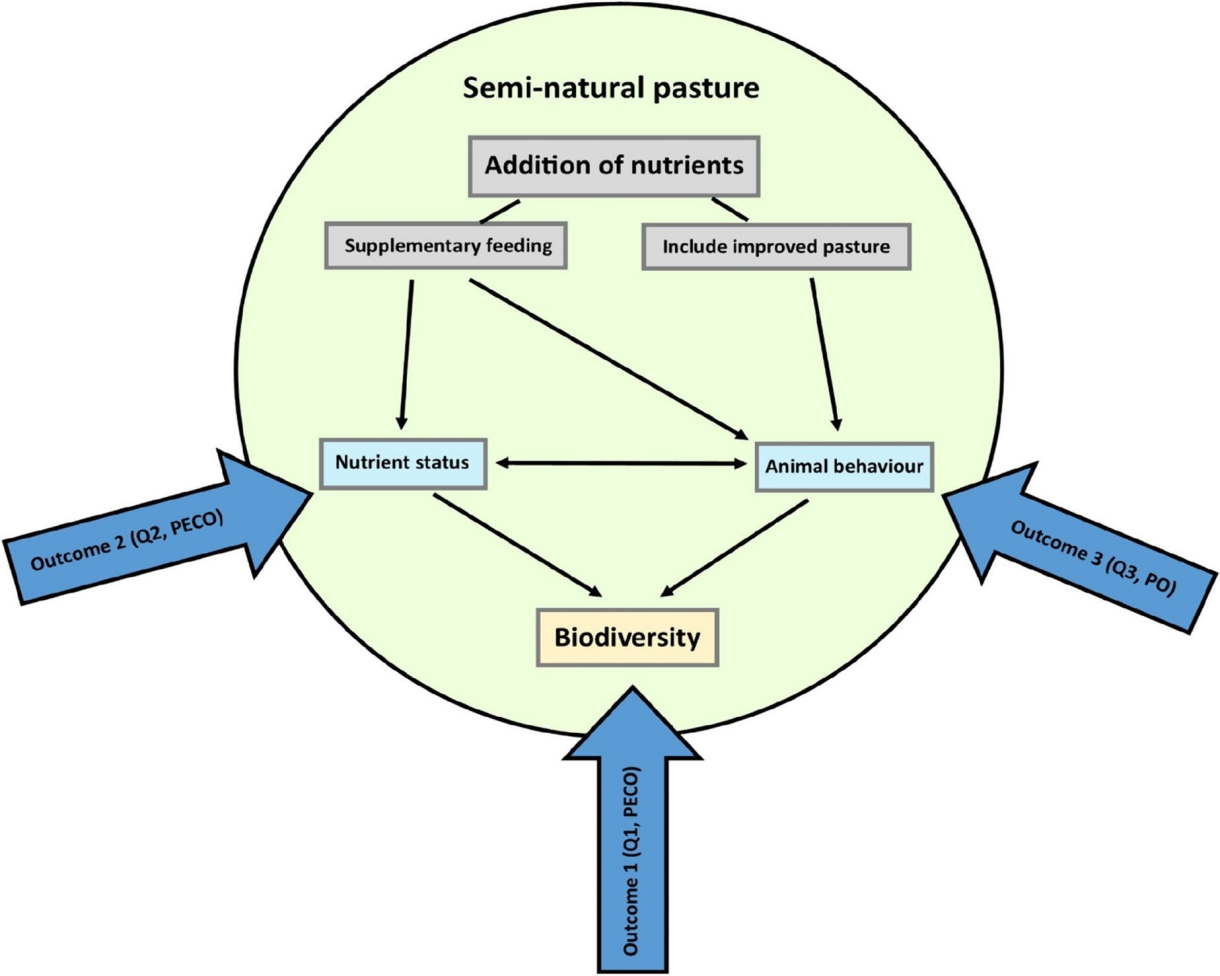
Conceptual model illustrating how nutrient addition might affect biodiversity in semi-natural pastures. Nutrient addition can occur either by supplementary feeding or by co-enclosing semi-natural and improved pastures within the same paddock. The thin arrows indicate possible impact directions. Note that supplementary feeding might impact the nutritional status either directly, via spillage, or indirectly, via manure and urine. The outcomes of the three respective questions (Q1, Q2, Q3) are structured within a P(EC)O framework indicated by thick arrows, to elucidate the relationships between the P(EC)Os. For definition of P(EC)Os, see Objective of the review

The mechanisms underpinning this review are illustrated by the conceptual model in Fig. 1. If grazers are given access to additional nutrient sources, there is a potential direct or indirect eutrophication risk of the semi-natural pasture that could impact biodiversity negatively. Direct eutrophication occurs in the form of fodder spillage. Indirect eutrophication occurs in the form of urine or manure. This may spread the additional nutrients over larger distances [23, 24], reflecting the behaviour of the grazing animals (e.g., regarding grazing habits, dietary choices, movements, defaecation and urination) [25, 26]. Studies have shown that supplementary feeding as well as co-enclosing semi-natural pastures with improved pastures may affect animal behaviour. For example, cattle often create so-called camping areas, where they rest for long periods of time. In such areas soil nutrients tend to accumulate [27]. Cattle have also been found to prefer grazing in productive nutrient-rich areas [28]. Due to the complexity of nutrient transport and its effects on biodiversity, animal movement does not necessarily mean that grazing animals transfer nutrients from the nutrient-rich areas to the semi-natural pastures if they are given access to such nutrient-rich areas within the same enclosure. Hence, these behaviours must be linked to measurements of their impact (Fig. 1). Furthermore, nutrient accumulation and grazing intensity depend heavily on, for example, grassland area, herd size, grazing season [29] and breed [30].

Many farmers in Sweden are dependent on financial subsidies to maintain semi-natural pasture management. Semi-natural pastures funded by the Common Agricultural Policy (CAP) programme 2014–2022 were expected to be managed according to a commitment plan, developed for each specific pasture by the local county administration board. The regulations formulated in the commitment plans aimed to promote biodiversity. Accordingly, giving grazers access to more nutrient-rich pastures by fencing the semi-natural pasture into the same enclosure as an improved pasture was often prohibited, as was supplementary feeding [21]. However, since 2023 the commitment plans linked to each specific semi-natural pasture have been replaced by general requirements related to the most crucial management aspects concerning regulations on supplementary feeding. These requirements differ between habitat types, meaning that the current regulations can be either stricter or more lenient for a specific pasture, compared to earlier regulations in the commitment plan.

The question has been raised whether eutrophication from supplementary feeding or co-enclosing nutrient-rich pasture areas with semi-natural pastures has enough scientific support to be considered in management regulations. Otherwise, strict regulations might not be necessary, and possibly even counterproductive [31]. Since the nutrient addition issue has not been sufficiently underpinned by scientific evidence, the Swedish Board of Agriculture called for a systematic review on the issue. Although the systematic review has been set up to be valid for a Swedish context, it should be of interest to stakeholders also in other countries.

Systematic reviews are designed to permit unbiased conclusions. Our review follows the guidelines for systematic reviews in environmental management issued by the Collaboration for Environmental Evidence [32]. The design of the review was established in detail in a peer-reviewed protocol [31]. Stakeholders representing the Swedish Board of Agriculture, Swedish Environmental Protection Agency, Swedish local county administration boards and the Swedish National Heritage Board commented on the protocol before submission, to ensure relevance to policy and practice.

The purpose of this systematic review was to provide information to support management of semi-natural pastures and protection of biodiversity. The primary question was: “What is the effect of giving grazers access to additional nutrient sources on biodiversity in semi-natural pastures?”.

## Objective of the review

The primary objective of this systematic review was to investigate whether giving the grazers of semi-natural pastures access to additional nutrient sources influences the biodiversity in the semi-natural pastures. The ultimate aim of the review was to facilitate evidence-based management of semi-natural pastures to promote biodiversity. To take the complex relationships visualised in Fig. 1 into account, we used three different review questions, one primary question and two supporting questions: “What is the effect of giving grazers access to additional nutrient sources on biodiversity in semi-natural pastures?” (Q1), “What is the effect of giving grazers access to additional nutrient sources on nutrient status of the soils of semi-natural pastures?” (Q2) and “How do the grazers of semi-natural pastures behave while having access to additional nutrient sources?” (Q3). The primary question (Q1) addresses how biodiversity is affected by additional nutrients in semi-natural pastures, either by co-enclosing the semi-natural pasture with more nutrient rich areas, or by supplementary feeding. The rationale behind the second question (Q2) is the assumption that a potential effect on biodiversity in the semi-natural pasture is driven mainly by eutrophication. The rationale behind the third question (Q3) is that a potential eutrophication of the semi-natural pasture is dependent on the behaviour of grazing livestock (Fig. 1). We applied no geographical restrictions when collecting and analysing the evidence.

The three questions were structured within a PECO framework as follows (the third question with a simplified PO-only structure):

The primary question (Q1, PECO) *What is the effect of giving grazers access to additional nutrient sources on biodiversity in semi-natural pastures?*:

Population: Semi-natural pastures.

Exposure: Giving the grazers access to (an) additional nutrient source(s).

Comparator: No additional nutrient source(s).

Outcome: Difference in biodiversity.

The first supporting question (Q2, PECO) *What is the effect of giving grazers access to additional nutrient sources on nutrient status of the soils of semi-natural pastures?*:

Population: Semi-natural pastures.

Exposure: Giving the grazers access to (an) additional nutrient source(s).

Comparator: No additional nutrient source(s).

Outcome: Difference in soil nutrient status.

The second supporting question (Q3, PO) *How do the grazers of semi-natural pastures behave while having access to additional nutrient sources?:*

Population: Grazers of semi-natural pastures, that also have access to (an) additional nutrient

source(s).

Outcome: Behavioural measures related to a possible nutrient relocation, grazing pressure or mechanical disturbance within the pasture.

The criteria are described in more detail below, in the section *Article screening and study eligibility criteria*.

## Methods

This systematic review was conducted according to a previously published protocol [31]. It follows the Collaboration for Environmental Evidence Guidelines [32] and conforms to the ROSES standards [33] (see Additional file 1).

### Deviations from the protocol

The few deviations from the protocol [31] are listed below, including justifications for the changes.

- We have revised Q1 and Q2 linguistically, to make them reflect our intentions more correctly. Q1 was formulated “*How does giving the grazers access to additional nutrient sources affect biodiversity in semi-natural pastures?”* in the protocol. It is now formulated “*What is the effect of giving grazers access to additional nutrient sources on biodiversity in semi-natural pastures?”*. Q2 was formulated “*How does giving the grazers access to additional nutrient sources affect the nutrient status of the soils of semi-natural pastures?”* in the protocol. It is now formulated “*What is the effect of giving grazers access to additional nutrient sources on nutrient status of the soils of semi-natural pastures?”*.
- Eligible populations
  - As eligible population we also included semi-natural forest and heathland pastures for Q1 and Q2, in addition to grass/forb dominated pastures, as these are also types of semi-natural pasture environments with high biodiversity values linked to extensive management. Consequently, as eligible population for Q3 we also included grazers of semi-natural forests and heathland pastures. The search strings (see Additional file 2) were adapted to identify also such studies. In addition, we have clarified the inclusion of pastures of natural origin in the eligibility criteria.
  - In the analysis, we did not separate the different types of semi-natural pastures or treated them differently. Due to the heterogeneity of the semi-natural grasslands, we have chosen to use grazer preference for the improved pasture areas instead of preference for the semi-natural pasture areas.
  - Clarification: We did not include studies on active herding systems because of our criterion on fenced pastures, which is rarely the case for herding systems, and because of the effect of herding itself on the grazer movement.
- Eligible outcomes
  - Studies targeting single species outcomes, including indicator species, were not included since our review is focused on overall biodiversity within and among organism groups (Q1).
  - The wording concerning potential outcome effects for Q1 and Q2 has been consistently revised to specify differences in outcomes (i.e. biodiversity levels and soil nutrient status), and not effects or changes.
  - Clarification: Grazing pressure-outcomes (Q3) were limited to nutrient/biomass removal or transportation.

### Search for articles

An exhaustive literature search for academic articles and grey literature was conducted in bibliographic databases and search engines, at websites of relevant organisations, and through snowballing and stakeholder contacts. Any type of publication will henceforward be denominated “article”.

#### Search terms and strings

Since the objective of this systematic review was defined by three questions and their respective P(EC)O structures, we developed three different search strings, one for each question. All information about the searches is provided in Additional file 2. This file includes database and platform information, how the search strings were adapted to the search capabilities and syntax of each specific database/platform, limits of the searches, date of searches, and the number of hits from each search. The search strings for Q1 and Q2 consisted of three search blocks, one with population terms, one with exposure terms and a final one with outcome terms. The search block with population terms (semi-natural pastures) and the search block with exposure terms (giving the grazers access to additional nutrient sources) were the same for Q1 and Q2, but the search block with outcome terms differed between the two search strings. Since the supporting question (Q3) is defined by population and outcome only, the search string for this question was restricted to two search blocks, one with population terms and the other one with outcome terms. In the Q3 search string, the search block with population terms was broader than that for Q1 and Q2, since the population of this question is grazing domestic animals in semi-natural pastures, that also have access to one or more additional nutrient sources. The broad population search block incorporates both the population terms (semi-natural pastures) and the exposure terms (giving the grazers access to additional nutrient sources) from Q1 and Q2. A search block with outcome terms (behavioural measures) was also added to the Q3 search string.

Only English search terms were used, except for in DiVA Portal and SwePub, where also Swedish was used. Since non-English articles most often have a title and abstract in English, English search terms capture articles written also in other languages. Articles written in English, Swedish, Danish, Norwegian, French, German or Spanish were taken into consideration.

The original search was performed in January 2021, in the seven bibliographic databases listed in Table 1. An updated search was performed in September 2023 in the same bibliographic databases, except for Directory of Open Access Journals (DOAJ). The primary reason for not searching in DOAJ in the updated search was that it was no longer possible to export the identified records from DOAJ in a format that can be imported into EndNote. Fortunately, these records are most likely found in searches in other sources, for example in Google Scholar. The same search strings (see Additional file 2) were used for both searches. Neither of the searches were limited as to publication date or publication type, according to Bramer and Bain [34].

**Table 1:** Bibliographic databases used to search articles

Database/platform	Search field	Language of search terms	Subscription information
Scopus	Title, Abstract, Keywords	English	Swedish Research Council Formas subscription
Web of Science Core Collection	Topic (search the fields: title, abstract and keywords)	English	Swedish Research Council Formas subscription includes: Science Citation Index Expanded; Social Sciences Citation Index; Arts & Humanities Citation Index; Conference Proceedings Citation Index-Science; Conference Proceedings Citation Index-Social Science & Humanities; Emerging Sources Citation Index
CAB Abstracts	Title, Abstract, Heading Words	English	Swedish Research Council Formas subscription on Ovid platform
Directory of Open Access Journals (DOAJ)*	All fields	English	Free, does not require a subscription
DiVA Portal	All fields	English and Swedish	Free, does not require a subscription
ProQuest Natural Science Collection	Title, Abstract, All subjects & indexing	English	Swedish Research Council Formas subscription includes: AGRICOLA; Agricultural Science database; Aquatic Sciences and Fisheries Abstracts*; Biological Science database; Biological Science index; Earth, atmosphere & Aquatic Science database*; Environmental Science database; Environmental Science index; Meteorological & Geoastrophysical Abstracts*
SwePub	All fields	English and Swedish	Free, does not require a subscription

Bibliographic databases used to search articles. Databases marked with an * are not included in the updated search

#### Bibliographic databases

The databases used are listed in Table 1.

#### Search engine

Searches were performed in the academic search engine Google Scholar on January 20, 2021, and September 18, 2023. It is not possible to use long search strings in Google Scholar, so we used three simple search strings in each of the languages English, Swedish, Norwegian and Danish, one for each P(EC)O (see Additional file 2). The search results were sorted by relevance and the first 50 results from each search string were exported from Google Scholar using Publish or Perish software [35].

#### Websites of relevant organisations

To find grey literature, we searched the websites of 51 relevant organisations, for example government agencies, environmental protection agencies, environmental research institutes, Swedish county administration boards, and (not peer-reviewed) journals. Simple search strings were used also for websites, and we searched in English, Swedish, Norwegian or Danish, depending on website. Search terms for each website, and the number of matching results, are provided in Additional file 2.

#### Supplementary searches

We looked through reference lists of relevant reviews, retrieved by the searches, to find additional articles (see Additional file 3). We also obtained some articles directly from stakeholders.

#### Estimating the comprehensiveness of the search

We used a list of benchmark studies to test the sensitivity of our searches (Additional file 4). The list contained seven references. All of those were retrieved by the searches, except from one grey report written in Finnish [36].

#### Assembling and managing search results

The results from each search (the original and the updated, respectively) were collated using the reference management software EndNote 20. Duplicates were removed using the de-duplication method described by Bramer et al. [37].

In order to remove records that were already identified and screened in the original search we used the method described by Bramer and Bain [34]. According to this method, the records from the original search and the updated search, respectively, are combined in the same EndNote library. After that, all duplicates (that is, both records of each duplicated reference) are removed. Most of the remaining records will then be records that have been added to the databases or search engines after the original search was performed. However, also records that were present in the original search, but not in the updated, will remain after duplicate removal. The reason for not finding these records in the updated search may be that indexes in databases, or rankings in search engines, have been updated since the original search was performed.

The number of hits reported in Additional file 2 are from the updated search, in the cases when such a search was performed.

### Article screening and study eligibility criteria

#### Screening process

Before the conclusive screening process started, a subset of articles (n = 350) was screened by five reviewers, based on title and abstract, with blinded decisions. Any disagreement was used to evaluate, and—if found necessary—more clearly define the eligibility criteria. This process was repeated until the criteria were interpreted and applied in a consistent way. Then the articles were screened for relevance in two stages. First, after removal of duplicates, they were single screened based on title and abstract. The reviewer had three options during the screening process: (1) include, (2) exclude, or (3) maybe include. Option 2 (exclusion) was applied only if it was completely obvious that the topic was out of scope. Articles coded with option 3 (n = 83) were screened by two other reviewers, with blinded decisions. Any disagreements were reconciled through discussion. In addition, in all 708 decisions were cross-checked by another reviewer (who already knew the decision of the initial screener).

Second, the full text of all the articles included in the first step were screened. This was done by two reviewers, with blinded decisions. Any disagreements were reconciled through discussion. A list of articles excluded in the second stage (full text) is provided, including reasons for exclusion (Additional file 5). Articles excluded in the first stage were not coded with a reason for exclusion.

Authors of the review were not allowed to assess the relevance of studies authored by themselves. All references, also those provided by stakeholders, underwent the same screening process.

#### Eligibility criteria

##### Q1 (PECO)

*Eligible population* Fenced, uncultivated, semi-natural pastures. Focus is on semi-natural pastures, i.e., grasslands that are the result of human management, and that require grazing by domestic animals to maintain their grass/forb domination and avoid being encroached by shrubs and trees. Studies on pastures of natural origin, i.e., grasslands grazed by domestic animals but mainly created and maintained by natural processes (such as fire or wildlife grazing) are also eligible, as well as semi-natural or natural forest and heathland pastures grazed by domestic animals. We make no distinction between these different types of origins of pastures and therefore include all in our main semi-natural pasture population term, hereafter called semi-natural pastures. There are no limitations as to geographic location of the pastures.

*Eligible exposure* Giving the domestic grazing animals access to one or more additional nutrient source(s). The grazing animals must be livestock present in Sweden, such as cattle, horses, sheep, and goats (pigs are excluded since they are not considered to be grazers). The additional nutrient source(s) may be in the form of supplementary feeding (the feeding site may be located outside, in the semi-natural pasture, or inside, in a byre), or by fencing the semi-natural pasture into the same enclosure as an improved pasture. The additional nutrient may not be added as fertilisers directly to the semi-natural pasture as, e.g., inorganic or organic manure.

*Eligible comparators* Eligible studies must include a control. The control site(s) must be semi-natural pasture(s) according to the population definition and not subjected of the above-described exposure.

*Eligible outcomes* Any outcome indicating a difference in biodiversity level, for example regarding measures of functional or taxonomic diversity, or vegetation structure. Single species outcomes are not eligible as our review is focused on overall biodiversity within and among organism groups.

*Eligible types of study design* Studies that quantify how giving the grazers of semi-natural pastures access to (an) additional nutrient source(s) affects the biodiversity values of the focal pastures. Comparisons can be made temporally and/or spatially, that is, ‘BA’ (Before/After), ‘CI’ (Control/Impact) as well as ‘BACI’ (Before/After/Control/Impact) and ‘RCT’ (Randomised Controlled Trial) designs are accepted.

##### Q2 (PECO)

*Eligible population* Fenced, uncultivated, semi-natural pastures. Focus is on semi-natural pastures, i.e., grasslands that are the result of human management, and that require grazing by domestic animals to maintain their grass/forb domination and avoid being encroached by shrubs and trees. Studies on pastures of natural origin, i.e., grasslands grazed by domestic animals but mainly created and maintained by natural processes (such as fire or wildlife grazing) are also eligible, as well as semi-natural or natural forest and heathland pastures grazed by domestic animals. We make no distinction between these different types of origins of pastures and therefore include all in our main semi-natural pasture population term, hereafter called semi-natural pastures. There are no limitations as to geographic location of the pastures.

*Eligible exposure* Giving the domestic grazing animals access to one or more additional nutrient source(s). The grazing animals must be livestock present in Sweden, such as cattle, horses, sheep and goats (pigs are excluded since they are not considered to be grazers). The additional nutrient source(s) may be in the form of supplementary feeding (the feeding site may be located outside, in the semi-natural pasture, or inside, in a byre), or by fencing the semi-natural pasture into the same enclosure as an improved pasture. The additional nutrient may not be added as fertilisers directly to the semi-natural pasture as, e.g., inorganic or organic manure.

*Eligible comparators* Eligible studies must include a control. The control site(s) must be semi-natural pasture(s) according to the population definition and not subjected of the above-described exposure.

*Eligible outcomes* Any outcome indicating a difference in soil nutritional status, for example regarding measures of nutrients in the soil, plant indicators (like Ellenberg values) or biomass production.

*Eligible types of study design* Studies that quantify how giving the grazers of semi-natural pastures access to (an) additional nutrient source(s) affects the nutritional status of the soils of the focal pastures. Comparisons can be made temporally and/or spatially, that is, ‘BA’ (Before/After), ‘CI’ (Control/Impact) as well as ‘BACI’ (Before/After/Control/Impact) and ‘RCT’ (Randomised Controlled Trial) designs are accepted.

##### Q3 (PO)

*Eligible population* Grazing domestic animals in semi-natural pastures or pastures of natural origin, that also have access to (an) additional nutrient source(s). We make no distinction between the different types of origins of pastures and therefore include all in our main semi-natural pasture population term, hereafter called semi-natural pastures. The grazing animals must be livestock present in Sweden, such as cattle, horses, sheep and goats (pigs are excluded since they are not considered to be grazers). The additional nutrient source(s) may be in the form of supplementary feeding (the feeding site may be located outside, in the seminatural pasture, or inside, in a byre), or an improved pasture within the same enclosure as the seminatural pasture. There are no limitations as to geographic location of the pastures.

*Eligible outcomes* Measures of animal behaviour related to (1) possible nutrient relocation within the pasture (for example, grazing habits, dietary choices, movements, and distribution of faeces and urine), or (2) grazing pressure in relation to nutrient/biomass removal or transport, or (3) mechanical disturbance (for example heavy trampling).

*Eligible types of study design* Studies that relate the focal behavioural measure to the access to (1) supplementary feeding, or (2) an improved pasture within the same enclosure as the semi-natural pasture. There must not be a comparator, although this would be preferable. That is, observational case studies are accepted, as well as studies of any kind of comparative design (‘BA’ [Before/After], ‘CI’ [Control/Impact], ‘BACI’ [Before/After/Control/Impact] or ‘RCT’ [Randomised Controlled Trial]).

### Study validity assessment

Studies that fulfilled the relevance criteria described above were subject to critical appraisal. We used CEECAT: Collaboration for Environmental Evidence Critical Appraisal Tool Version 0.2 (prototype) [38] as a basis, to formalise our assessments and make them more transparent and replicable. In accordance with CEECAT, the studies were categorised as having low, medium or high risk of bias. All validity decisions were performed by two authors independently, to ensure consistency. Any disagreement was discussed by the two reviewers to reach consensus. Authors of the review were not allowed to perform critical appraisal of their own work. The final validity decisions are provided in Additional file 6.

Relevant studies were included in the review even though they were assessed to be of high risk of bias, but study validity was taken into consideration in the analyses. This was done by conducting a quantitative sensitivity analysis, as well as in the overall grading of evidence (i.e., the assessment of the strength of evidence of the research base as a whole in relation to the respective review questions). Study validity was one of several aspects forming the basis for this assessment, see Data synthesis and presentation for other aspects. However, data only allowed us to perform quantitative analysis on the effects on grazer behaviour (Q3), see Data synthesis and presentation.

### Data coding and extraction strategy

Data were compiled using a spreadsheet file (see Additional file 7). All reviewers participated in the data extraction. Data from at least 25% of the articles allocated to each reviewer were cross-checked by another reviewer, to ensure consistency. In addition, one reviewer checked all extracted data for consistency prior to analysis, and clarification needs were resolved by discussion within the reviewer group.

Concerning quantitative data, outcome means and measures of variability (standard deviation) or precision (standard error) were extracted from tables, graphs and text in the included articles. When necessary, image analysis software (WebPlot-Digitizer [39]) was used. Extracted outcomes included measures of species richness, species diversity (e.g. diversity indices) and abundance of taxonomic or functional groups of organisms (Q1), measures of soil nutrient status or biomass (Q2), and observational data on grazing behaviour related to their use of the pastures or measures of their nutrient intake (Q3).

When feasible, data were recorded in the main spreadsheet as they were reported in the articles, and transformations and calculations were mainly performed at the analysis stage. Most of the behaviour studies (Q3) reported outcome data as a relative index of grazing preference for different types of vegetation or areas within pastures. Hence, for studies not reporting such behaviour data in a similar manner, we made a first extraction to a separate file, where index values were calculated and transferred to the main database. These outcomes were recorded as either “relative preference” (RP) or “electivity” (E); see below for index calculations. Similarly, data extracted from figures were also extracted in a separate file prior to inclusion in the database.

Where relevant outcomes had been reported in a format that impeded inclusion in quantitative analyses, study authors were asked to supply raw or summarised digital data instead, and/or provide clarifications if needed. Metadata, such as data on potential effect modifiers (see below), were extracted if present in the published material; no requests were sent for unpublished metadata. Some metadata were carefully derived from other studies, where study setup was obviously shared between studies. Requested data that were received from authors did not include any eligible data, rather confirmed the non-eligibility of the study, and hence no additional data were added to the main data sheet. However, two authors provided clarifications concerning a set of overlapping studies, which made it possible to select appropriate data for our analyses (see Additional file 7). Studies with overlapping data were included in the file as separate studies, but specified as completely or partly redundant, and if needed merged in the analysis (see Data synthesis and presentation).

If quantitative data were not presented in the study, qualitative outcomes were extracted. To the extent possible, the study authors’ own qualitative descriptions of the results were extracted, otherwise the reviewer interpreted the results and described them qualitatively.

#### Potential effect modifiers/reasons for heterogeneity

To the extent that data were available, the following potential effect modifiers were recorded:

- Climate zone
- Landscape type
- Pasture type (semi-natural or natural)
- Soil type
- Area of semi-natural pasture
- Area of improved pasture
- Habitat structure
- Vegetation/Habitat type
- Nutritional status
- Adjacent habitats
- Landscape context
- Time since inclusion of improved pasture
- Management history
- Kind of supplementary feed
- Amount of supplementary feed
- Feeding frequency
- Location of feeding site
- Species of grazers
- Breed of grazers
- Age of grazers
- Sex of grazers
- Number of individuals per unit of area

The possible effect modifiers were identified through discussions by the experts in the review team; some of them were also suggested by stakeholders. They were considered to be the main reasons for heterogeneity in the review, covering key aspects of management and environmental factors affecting biodiversity in semi-natural pastures, including the broader spatio-temporal context.

For data that were pooled across pastures, average values as well as minimum and maximum values for pasture areas and animal densities were extracted, if possible. When climate zone data were not available in the included articles, we retrieved them from Beck et al. [40], using the coordinates or specified locations of study sites.

### Narrative synthesis

Each of the included articles is summarised in an overview table presenting the study setup, which review question(s) it targeted, a short summary of the result(s) and the risk of bias that has been critically appraised by the review team. We used EviAtlas [41] to create an interactive evidence atlas, with information about the first author, publication year, targeted review question(s) and type of nutrient addition for each included article (Additional file 8). In addition to these summaries, study findings are briefly summarised in text.

### Data synthesis and presentation

The studies included in this review reported effects of either co-enclosure of an improved pasture area or supplementary feeding on outcomes related to all the three review questions described above. It was, however, only for the effects on grazer behaviour (Q3) that data allowed for quantitative analyses, as there was no between-study replication of specific exposure-outcome combinations neither for Q1 nor for Q2. Concerning the impact of co-enclosing an improved pasture, the reported outcomes included time spent on different vegetation types or diet composition, but only measures related to time spent were replicated across studies and hence included in our analyses. In addition, diet composition was rarely directly related to the different vegetation types of the pasture because of overlapping plant species composition among the vegetation types.

Most studies reported a calculated preference index based on time spent on each vegetation type, either as *relative preference (RP)*:

$$R{P}_{i}=\frac{{B}_{i}}{{A}_{i}}$$

or *electivity (E)*:

$${E}_{i} = \frac{\left({B}_{i}-{A}_{i}\right)}{\left({B}_{i}+{A}_{i}\right)}$$

where A = the proportion of vegetation type *i* within the paddock and B = the proportional time spent within vegetation type *i.* The equations lead to that each index can be calculated directly from the other:

$$R{P}_{i}=\frac{\left({E}_{i}+1\right)}{\left(1-{E}_{i}\right)}$$

$${E}_{i}=\frac{\left(R{P}_{i}-1\right)}{\left(R{P}_{i}+1\right)}$$

Quantitative analyses on the effects of including an improved pasture area were limited to the two preference measures *RP* and *E*. Although none of the studies with data on these measures used a control pasture (a semi-natural pasture not co-enclosed with an improved area), they were included in the analyses. The preference measures are by definition relative to the area covered by each vegetation type, hence the values 1 (for *RP*) and 0 (for *E*), respectively, can be seen as expected outcomes given that there would be equal preferences for all vegetation types. Deviation from this expected outcome can be seen as a sign of preference (RP > 1; E > 0) or avoidance (RP < 1; E < 0) for the given vegetation type.

For consistency purposes, we used e*lectivity* (*E*) for the improved area in the pasture as response variable in our analysis. The characteristics of the non-improved semi-natural or natural areas differed between studies, as did the level of detail of reporting vegetation types in those areas (see Additional file 7). As most included studies focused on the behaviour of adult animals, we excluded data on their offspring in our data synthesis (cf. [42]).

Concerning the effects of supplementary feeding, the only replicated comparable outcome measure across studies was pasture dry matter intake (DMI). DMI was reported in different units (total, average per animal, average per day, etc.); hence, we used the log-ratio between supplemented and non-supplemented pastures/animals as response variable in the analysis.

Due to the lack of reported measures of variability (only available for 26% of the studies in total, and 14% of the Q3 studies), we were not able to conduct a meta-analysis. Instead, a simplified approach based on mixed effects modelling was used, using the packages *nlme* [43], *emmeans* [44] and *car* [45] in R [46]. The quantitative analyses were restricted to the following analytical setups for Q3:

1. Estimating the effect of co-enclosing an improved pasture area within the paddock on *electivity (E)* of the improved pasture area, and how those effects depended on different *grazer species*, whether or not the herd was mixed with other grazer species *(mixed herd*)*, proportion of improved pasture area*, total animal density (livestock units: *LSU* [47, 48]) and *outcome type* (grazing, resting, general). In the analysis, the *LSU* variable was limited to adult animals only, as that was the only measure consistently reported across studies (see Additional file 7 for details on the LSU measure). The datasets were reduced depending on which variables were included. Other effect modifiers were not considered applicable in the analysis because they were not reported consistently across studies.
  Given the limited amount of available and recorded data, we used a reduced approach to a stepwise model construction based on available data for the variables of interest: (1) one overall model to test how *grazing E* was affected by *grazer species* and *proportion of improved pasture area* as fixed effects and *study ID* as random effect; (2) an extended model including the interaction between *grazer species* and a *mixed herd* variable (mixed/not mixed); (3) another extended model testing the marginal effect of *LSU* on the grazing preferences (including only studies with data on *LSU*); and (4) a reduced cattle only-model testing the marginal effect of *outcome type* in a model including the statistically significant variables from the previous models (there were no other outcome types than grazing for the other grazer species).
2. Estimating the effect of supplementing the grazing animals with additional nutrients on their forage intake, measured as *dry matter intake (DMI)*.
  We used a mixed null model, with only the number 1 as explanatory variable, to test whether the log ratios were statistically significantly different from 1 or not, including *study ID* as random factor. Given the low number of studies (5) and different setups and conditions across studies, no further analysis was conducted.

All analyses were weighted using the square root of the number of spatial replicates within each study, i.e. the number of pastures. For setup 2, analysing the effects on forage intake, the square root of the total number of pastures in the comparison (i.e. treatment vs. control) was used as weighing factor. Temporal replication (data from > 1 year) was dealt with using the mixed model approach with year as random factor.

Statistical significance of the models was estimated using Type II-tests (package *car* [45]) and confidence intervals from contrast analysis (package *emmeans* [44])*.*

A sensitivity analysis was conducted by reducing the above-described models by excluding data from studies with a high risk of bias. Complete and reduced data models were compared and discussed qualitatively.

We were not able to examine the possible influence of publication bias on the synthesis because of the incomplete reporting of variation, precision and statistical significance in the included studies.

## Review findings

### Review descriptive statistics

**Fig. 2:**
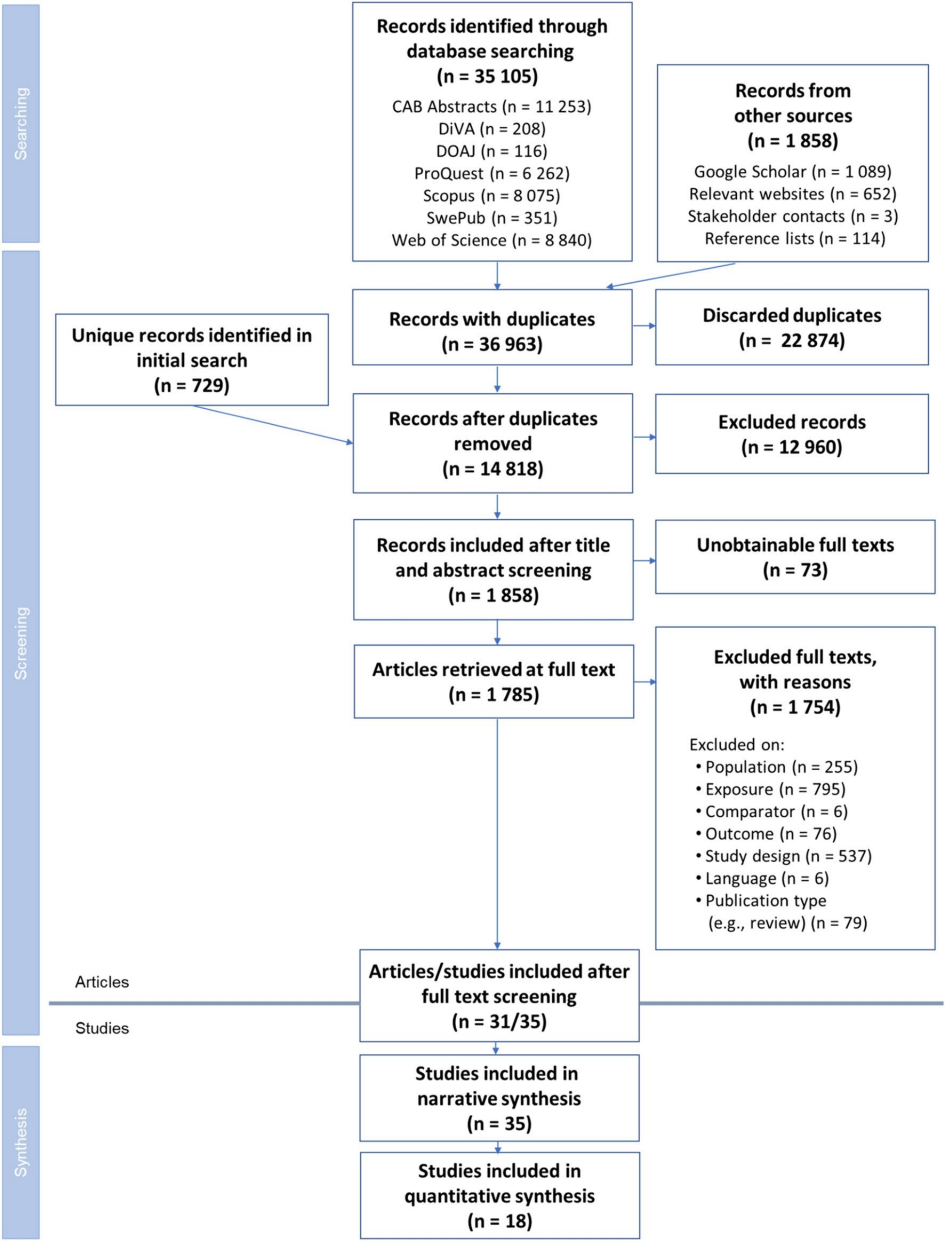
Flow diagram describing the steps in the searching and screening process. Note that an article may exhibit several reasons for exclusion, and in these cases only one of the reasons is counted. Hence the allocation between reasons for exclusion only provide a hint. After ROSES Flow Diagram for Systematic Reviews [49]

The database search for published literature resulted in a total of 35 105 articles (Fig. 2). Most were found in CAB abstracts (11 253), followed by Web of Science (8 840), Scopus (8 075) and ProQuest (6 262). Screening through other sources, for example Google Scholar, resulted in 1 858 articles. In all, 36 963 records were identified before duplicate removal. After removing duplicates there were 14 818 records, including 729 unique records that were only identified in the original search (see section Assembling and managing search results). After screening on titles and abstracts, 1 858 articles remained. 73 articles were unobtainable in full text (Additional file 9), meaning that 1 785 articles were retrieved at full text. The number of articles included after full-text screening was 31. These are listed in Table 2 and Table 3. Reasons for exclusion are provided in the Additional file 5.

**Table 2:** Overview of included articles/studies concerning the effects of co-enclosing semi-natural pasture(s) with an improved pasture

Article	Setup (relevant for this review)	Q	Results (relevant for this review)	Risk of bias
Andrée 2011* [28]	Four-year study of cattle grazing, resting and urination/defaecation behaviour in 2–9 semi-natural Nordic pastures in Uppsala, Sweden, including improved areas of grassland (median: 30% of the area)	3	In general, cattle had a relative preference (1) for both grazing and urination/defaecation in more nutrient rich areas of the pastures, and the highest preference was for the previously improved areas. Preference for improved areas were statistically significantly (2) different from dry areas all years concerning all behaviours (with resting behaviour varying across years), whereas other differences were not consistently statistically significant	MEDIUM
Benavides 2009* [55]	Six-year study of cattle, sheep and goat alone, and mixed herds with cattle or sheep with goats, grazing behaviour in eight cleared heathland pastures in Asturias, Spain, including improved areas of grassland (one third of the area sown with ryegrass and white clover). Four years of data on behaviour	3	Data indicate (3) that cattle and sheep had a relative preference (1) for improved pasture areas, both alone and when in the same paddock as goats. Goats also preferred the improved areas when alone but, in contrast, they showed a relative preference for the cleared heathland when together with either cattle or sheep during three out of 4 years	MEDIUM
Celaya 2007*^a^ [60]	Two-day study of cattle, sheep and goat grazing behaviour, grazing as a mixed herd on a highland heather-gorse pasture with improved areas (21% of the area sown with ryegrass and white clover) in Asturias, Spain. Study design and study area as in Celaya 2008 except for the exact proportion of improved area	3	Data indicate (3) that all grazer species had a relative preference (1) for improved pasture areas, but goats had a less pronounced relative preference compared to cattle and sheep	MEDIUM
Celaya 2008*^a^ [61]	Six-month study of cattle, sheep and goat grazing behaviour, grazing as a mixed herd on a highland heather-gorse pasture with improved areas (24% of the area sown with ryegrass and white clover) in Asturias, Spain. Study design and study area as in Celaya 2007 except for the exact proportion of improved area	3	Data indicate (3) that all grazer species had a relative preference (1) for improved pasture areas, but goats had a less pronounced relative preference compared to cattle and sheep	MEDIUM
Clarke 1995* [58]	One-year study of sheep (and deer) grazing preference in six partly improved upland heathland pastures (one sixth of the area being fertilised and sown with a *Agrostis* sp. and *Festuca* sp. mix) in north-east Scotland. Improvements were made in three different spatial pattern arrangements within each pasture	3	Data indicate (3) that sheep had a relative preference (1) for the improved areas in all pastures and periods of the study	MEDIUM
Ferreira 2012* [62]	Four-month study of horse grazing behaviour, grazing a highland heather-gorse pasture with improved areas (24% of the area sown with ryegrass and white clover) in Asturias, Spain	3	Data indicate (3) that horses had a relative preference (1) for the improved pasture areas but showed relatively less preference for improved areas in September compared to June	MEDIUM
Ferreira 2013*^b^ [63]	One-year study of cattle, horse, sheep and goat grazing behaviour, grazing as a mixed herd on a highland heather-gorse pasture with improved areas (24% of the area sown with ryegrass and white clover) in Asturias, Spain. Study design seems to be identical to Osoro 2005, but months with presented data partly differ	3	Data indicate (3) that cattle, horses and sheep had a relative preference (1) for grazing in improved areas of the pasture. In contrast, goats grazed in general less on those areas, and showed a relative preference for non-improved areas in two out of six months of the study	MEDIUM
Ferreira 2017* [56]	Three-month study of cattle and goat grazing behaviour, grazing a highland heather-gorse pasture with improved areas in Asturias, Spain. Study included one paddock with only cattle (69% improved) and one with both cattle and goat (25% improved). Improved pasture areas were sown with ryegrass and white clover	3	Data indicate (3) that cattle had a relative preference (1) for the improved pasture areas, but this was less pronounced when grazing alone in the paddock with 69% of improved pasture area compared with grazing together with goats in the paddock with 25% of improved pasture area. Goats had in general less relative preference for improved areas compared to cattle, which was negatively associated with the preference values for cattle (comparing the two months)	MEDIUM
Hester 1996* [59]	One-year study of sheep grazing behaviour, alone or in a mixed herd with deer, on a partly improved upland heather moorland pasture in three pastures in Glensaugh, Scotland (50% of the area reseeded with mainly *Lolium perenne* and *Agrostis capillaris*)	3	Data indicate (3) that sheep had a relative grazing preference (1) for the improved areas, both alone and together with deer	MEDIUM
Kaufmann 2013* [57]	Two-year study on general occupancy behaviour of cattle on three mixed woodland-grassland highland pastures (single herd, moved between pastures) in Alberta, Canada, including modified lowland grassland (average: 3% of the area)	3	Cattle had a statistically significant (2) relative occupancy preference (1) for the improved areas, which was also statistically significantly (2) higher compared to the other habitats within the pasture	MEDIUM
López-López 2015 [64]	Two-year study of two sites, but data on grazing behaviour of a mixed herd of cattle and horses from one of the partly improved (25% of the area sown with ryegrass and white clover) highland heather-gorse pastures in Asturias, Spain. Redundant data (cf. López-López 2019)	3	Data indicate (3) that cattle and horses had a relative preference (1) for grazing in the improved areas in all studied seasons of the year	HIGH
López-López 2019* [42]	Two-year study of two sites, but data on grazing behaviour of a mixed herd of cattle and horses from one of the partly improved (25% of the area sown with ryegrass and white clover) highland heather-gorse pastures in Asturias, Spain. Study design seems to be identical to López-López 2015, but more clearly explained here	3	Data indicate (3) that cattle and horses had a relative preference (1) for grazing in the improved areas in all studied seasons of the year. The relative preference was more pronounced in June 2012 compared to September 2012 and August 2013	MEDIUM
Osoro 2005*^b^ [65]	One-year study of cattle, horse, sheep and goat grazing behaviour, grazing as a mixed herd on a highland heather-gorse pasture with improved areas (24% of the area sown with ryegrass and white clover) in Asturias, Spain. Partly redundant data (cf. Ferreira 2013)	3	Data indicate (3) that cattle, horses and sheep had a relative preference (1) for grazing in improved areas of the pasture. In contrast, goats grazed in general less on those areas, and showed a relative preference for non-improved areas in one out of six months of the study	MEDIUM
Pelve 2007 [66]	One-year study of cattle grazing behaviour in 3 semi-natural Nordic pastures in Uppsala, Sweden, including improved areas of grassland (average: 34% of the area). Redundant data (cf. Andrée 2011)	3	Data indicate (4) that cattle had a relative preference (1) for grazing in improved areas	MEDIUM
Pelve 2008 [67]	One-year study of cattle grazing behaviour in 3 semi-natural Nordic pastures in Uppsala, Sweden, including improved areas of grassland (average: 34% of the area). Redundant data (cf. Andrée 2011)	3	Data indicate (4) that cattle had a relative preference (1) for grazing in improved areas	MEDIUM
Pelve 2010 [68]	One-year study of cattle grazing behaviour in 9 semi-natural Nordic pastures in eastern-central Sweden, including improved areas of grassland. Redundant data (cf. Andrée 2011)	3	Cattle had a statistically significantly (2) higher relative preference for grazing and urination/defaecation in the improved areas compared to the non-improved areas of the pastures	MEDIUM
Pelve 2020 [69]	Two-year study of cattle grazing, resting and urination/defaecation behaviour in 2/9 (first/second year) semi-natural Nordic pastures in Uppsala, Sweden, including improved areas of grassland (median: 30% of the area). Redundant data (cf. Andrée 2011)	3	Data show a general tendency for cattle to have a relative preference (1) for grazing, resting and urination/defaecation in more nutrient rich areas of the pastures. The preference index for improved areas was statistically significantly (2) different from all but the mesic areas both years and for all behaviours, except for grazing wet areas the first year, but not statistically significantly (2) different from mesic areas in all cases except urination behaviour the second year	MEDIUM
Takala 2015 [24]	Two-year study on biodiversity in cattle grazed pastures, comparing 18 semi-natural forest pastures connected to fertilised grassland areas with 7 semi-natural forest pastures without such connection, in Karelia, Finland	1	Pastures connected to improved areas had lower plant and bryophyte species richness as well as plant diversity, but higher cover of bryophytes, but differences were statistically significant (2) only for the plant species richness and diversity measures. Note: data are from 1 year for each pasture category, but different years (covering a period of 3 years)	LOW
Uytvanck 2010* [29]	One-year study on cattle occupancy and grazing behaviour on one mixed habitat semi-natural pasture including 60% former agricultural land in northern Belgium	3	Data indicate (3) that cattle had a relative preference (1) for the former agricultural land, both concerning general occupancy and grazing specifically. This preference tended to be higher compared to other habitats except for wooded pasture areas in summer (similar) and winter (wooded pasture more often used for foraging). Nitrogen intake in grassland and wooded pasture was comparable, but negligible in forest. Nitrogen excretion was on average 92.5% of the intake, and the redistribution was related to the intake pattern	HIGH

Setup, Review question number (Q), Results, and Risk of bias for studies investigating the effects on the three outcome categories (see column Q) of including an area of improved grassland in the same paddock as the semi-natural grassland. Numbers/asterisks/letters indicate: (1) Relative preference: indicates how the grazers select certain types (habitat, vegetation, etc.) of the pastures in relation to its areal coverage. In the quantitative analysis of this review, we have used the relative preference for improved areas of the pasture, for consistency across studies, estimated as electivity. See Methods for details. (2) Statistically evaluated by authors, concerning the relationship(s) described. In complicated cases, with many comparisons, results are here briefly summarised. (3) Not statistically evaluated by authors, concerning the relationship(s) described. (4) Statistically evaluated by authors, but results are unclear concerning statistical significance. (*) The article contains a study that was included in the quantitative analysis of Q3. (a)/(b) The included study was considered part of the same study in the quantitative analysis as the other study marked with the same letter. Details on validity decisions are provided in Additional file 6

**Table 3:** Overview of included articles/studies concerning the effects of supplementary feeding in semi-natural pastures

Article	Setup (relevant for this review)	Q	Results (relevant for this review)	Risk of bias
Avondo 2002 [70]	Eight-year study with data from nine feeding trials with data on the effect of in-house (in pen) supplementation on forage intake of sheep grazing semi-extensive pasture systems on Sicily, Italy. There are no data on number of pastures, just that there were 670 forage intake data from 210 animals	3	The provision of a supplement caused a reduction in pasture intake in pastures. Statistically significant (2) model improvements by including crude protein (CP) of supplement as a predictor, not dry matter (DM) of supplement. Separate results for pastures with less than or equal to, or more than, 16% CP content	High
Bowman 1999* [71]	Experiment 2: Three-month study on the effect of two supplementary feeding methods (same supplement formulation) on cattle forage intake on native range pastures, among other variables, in Montana, USA	3	Both feeding methods resulted in statistically significantly (2) higher forage intake compared to the control group. There was no statistically significant (2) difference between the feeding methods	Medium
Clariget 2016* [72]	One-year study of the effects of different types/combinations of supplementation on dry matter intake (DMI) of forage by cattle grazing a natural pasture in Cerro Largo, Uruguay	3	All treatments resulted in lower DMI compared to the non-supplemented control, with the lowest DMI for cattle fed crude glycerin, which was the only DMI estimate statistically significantly (2) lower than the control group	High
Da Ronch 2005 [50]	Two-year study on the effects of concentrate supplementation on plant species richness in three pairs of near-alpine (plateau) pastures in Vicenza, Italy, grazed by cattle	1	Data indicate (3) that on average the pastures where cattle were fed supplements, the species richness was higher. However, that difference seems to be driven by a big difference in species richness between the third pair of pastures	High
Guerrero 2018* [73]	Three-month study on the effect of supplementation with either sugarcane and urea or sugarcane only on sheep forage intake on natural pastures in Huambo, Angola	3	Non-supplemented sheep consumed statistically significantly (2) more forage than supplemented sheep. Among the supplemented sheep, the group supplemented with sugarcane and urea consumed statistically significantly (2) less forage than the group supplemented with sugarcane only	High
Mosley 2017* [51]	Two-year study of the effect of barley-based commercial sheep pellet supplement, compared to salt supplement only, on forage intake by sheep, plant community composition and plant yield in a highland rangeland in Montana, USA. Sheep were handfed in paddock. Data from two periods in summer	1	There were no statistically significant (2) differences in the plant community in the first period. In the second period, there were statistically significantly (2) more perennial graminoids and less other forbs in the plant community where sheep were supplemented	Medium
		2	The only statistically significant (2) difference in plant yield was that there were less other forbs where sheep were supplemented in the second year (measured separately for perennial graminoids, annual grasses and other forbs)	Medium
		3	Supplemented sheep statistically significantly (2) preferred annual grasses and avoided perennial graminoids in the first period, but avoided annual grasses in the second period, when non-supplemented sheep avoided perennial graminoids (no between-group test). There were no statistically significant (2) differences in dry matter intake in any period	Medium
Niemelä 2008 [52]	One-year study on the effect of supplementation of cattle (creep feeding of either flattened oat as the supplement or a commercial concentrate) on the nutritional status of the vegetation of four semi-natural coastal meadows along the Bothnian Bay, Finland	2	Data indicate (3) that the four meadows, two with supplementation and two without, differed in their vegetation nutrient status (P, K, Ca, Mg, S, Na, Fe, Zn, Cu, Mn). They also belonged to different farms, and hence locations. For details on the differences, see Table 2 in article	High
Ormaechea 2021 [74]	Two-year study of the effects of supplement blocks on sheep use of two steppe pasture paddocks in Santa Cruz, Argentina. GPS tracking was used to estimate animal movements before and after supplement blocks were placed in the two pastures	3	Sheep spent statistically significantly (2) more time in the area around the target sites and used a statistically significantly (2) larger proportion of the paddock area when supplement blocks were present	High
Souza 2023 [75]	Three-month study on the effect of supplementation on sheep movement on Caatinga rangeland in Pernambuco, Brazil. Nutrient intake also studied but interpreted as total intake (including supplement). The study was conducted in three periods, from rainy to drought period	3	Supplemented animals showed statistically significantly (2) lower concentration in their movement (lower Kernel density) compared to non-supplemented animals, especially in the rainy period	High
Sowell 2003* [76]	Two-year study on the effect of two supplementary feeding methods (same supplement formulation) on cattle forage intake on six native range pastures, among other variables, in Montana, USA	3	Supplemented cattle had statistically significantly (2) higher forage dry matter intake than non-supplemented cattle, both concerning dry matter intake and dry matter intake per kg body weight, in both years. A restricted supplement method resulted in the highest intake values	Medium
Yang 2020 [53]	Four-year study on the effect of supplementation on nutrient cycling, focusing on carbon (C), in six alpine pastures grazed by yak in Gansu province, China. Yaks were fed oat hay in overnight shelters	2	There were statistically significantly (2) higher litter C return and forage biomass in the grazing system with oat hay supplementation	Medium
		3	Conclusions from the structural equation model states (4) that yaks supplemented with oats hay increased the C return from litter and dung, thereby enhancing forage biomass in the next year	Medium
Yang 2021 [54]	Four-year study on the effect of supplementation on nutrient cycling, focusing on nitrogen (N), in six alpine pastures grazed by yak in Gansu province, China. Yaks were fed oat hay in overnight shelters	2	There were statistically significantly (2) higher litter N return and forage biomass in the grazing system with oat hay supplementation, but there was no statistically significant (2) effect on soil N stock	Medium
		3	Authors conclude that soil N stock was relatively insensitive to grazing intensity influenced by supplementation with hay	Medium

Setup, Review question number (Q), Results and Risk of bias for studies investigating the effects of supplementary feeding on the three outcome categories (see column Q). Numbers/asterisks indicate: (2) Statistically evaluated by authors, concerning the relationship(s) described. In complicated cases, with many comparisons, results are here briefly summarised. (3) Not statistically evaluated by authors, concerning the relationship(s) described. (4) Statistically evaluated by authors, but results are unclear concerning statistical significance. (*) The article contains a study that was included in the quantitative analysis of Q3. Details on validity decisions are provided in Additional file 6

### Mapping the quantity of articles relevant to the review questions

**Fig. 3:**
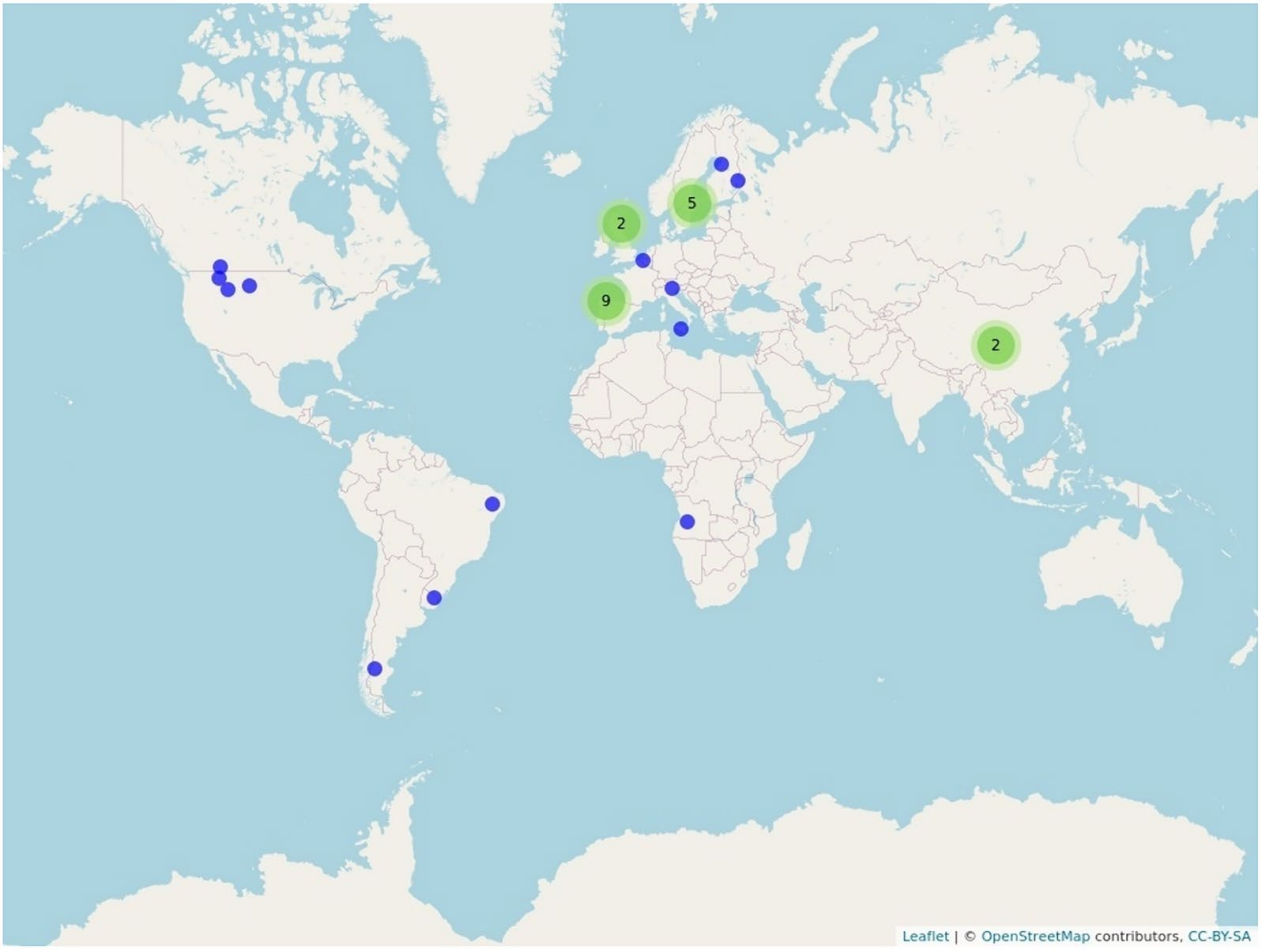
Geographical distribution of the included articles. Green circles indicate that several articles (the number within the respective circle) were based on studies performed in the same location. Use the interactive atlas (Additional file 8) to zoom into the map and resolve each specific article

The studies in about two thirds of the articles (21 out of 31) included in the review were conducted in Europe. Seven of the articles were from North and South America, two were from Asia, and only one article was from Africa (see Fig. 3 and Additional file 8). The dominance of European articles is mainly due to the large number of articles (14) published by two research teams based in Spain (9 articles) and Sweden (5 articles), each of which included partly overlapping data. Among these, three of the Spanish and four of the Swedish articles were published as grey literature. One more article was published as grey literature, whereas the rest of the included articles (23) were published in scientific journals.

### Narrative synthesis including study validity assessments

Twelve of the 31 included articles analysed effects of supplementary feeding and 19 analysed effects of including improved grassland in the same enclosure as semi-natural grassland (Tables 2, 3).

Three of the 31 articles investigated two or three of the review questions (P[EC]Os). Each P[EC]O in these articles is defined as a separate “study”. Accordingly, there are 35 studies within the 31 included articles. Nine of the studies had a high risk of bias, 25 had a medium risk, and one had a low risk of bias. Detailed information on each included article is presented in Additional file 7.

For the primary question Q1, only three studies were found that fulfilled our criteria, described in Takala et al. [24], Da Ronch et al. [50], and Mosley et al. [51]. Takala et al. was the only study that investigated effects on biodiversity of co-enclosing semi-natural grasslands with an improved pasture area. The two other studies investigated effects of supplementary feeding on plant species richness. The study described in Da Ronch et al. was judged as having a high risk of bias (Tables 2, 3).

For the supporting question Q2 we found four studies that fulfilled our criteria, described in Mosley et al. [51], Niemelä et al. [52], Yang et al. [53] and Yang et al. [54]. All of them addressed the effects of supplementary feeding on the nutrient status of the soils of semi-natural pastures (Table 3).

Most studies were related to the supporting question Q3. In total, we found 28 studies that fulfilled our criteria, of which 18 investigated grazing animal behaviour related to the co-enclosure of an improved pasture, and ten investigated animal grazing behaviour related to supplementary feeding (Tables 2, 3).

Thirteen articles contained unique quantitative data on grazing behaviour in pastures where semi-natural and improved pasture areas were co-enclosed in the same paddock(s). Among these 13 articles, one pair of articles included partly overlapping data and one pair of articles shared identical study setups and area but reported data from two different years. Therefore, only eleven unique studies were used in the analysis of Q3. Ten of these studies included behaviour related to the choice of grazing areas, and one study included resting and urination/defaecation behaviour in addition to the grazing behaviour. Two studies included the general behaviour/choice of area within the pasture, of which one study presented those data in contrast to data on grazing behaviour specifically.

Eight studies included cattle as grazer species and five of these studied paddocks with cattle only. Five studies included sheep, of which three had paddocks with sheep only. Four studies included goats, but only one included paddocks with goats only. Similarly, three studies included horses, but only one of those included paddocks with horses only. The only replicated single grazer species and outcome measure combinations across the included studies were cattle grazing behaviour (4 studies, described in Andrée et al. [28], Benavides et al. [55], Uytvanck et al. [29], Ferreira et al. [56]), cattle general behaviour (2 studies, described in Uytvanck et al. [29] and Kaufmann et al. [57]) and sheep grazing behaviour (3 studies, described in Clarke et al. [58], Hester et al. [59], Benavides et al. [55]) The only combination of grazer species that was replicated across studies was cattle and goats grazing the same paddock(s), found in two studies (described in Benavides et al. [55] and Ferreira et al. [56]).

### Data synthesis

There were not enough literature and data to perform any quantitative analysis related to Q1 or Q2. Therefore, it is not possible, based on this systematic review, to draw any conclusions concerning if supplementary feeding or co-enclosing semi-natural and improved pastures affect biodiversity or the nutritional status in semi-natural pastures. However, indirect effects based on animal behaviour (Q3), had enough data to be compiled and analysed, as described above.

**Fig. 4:**
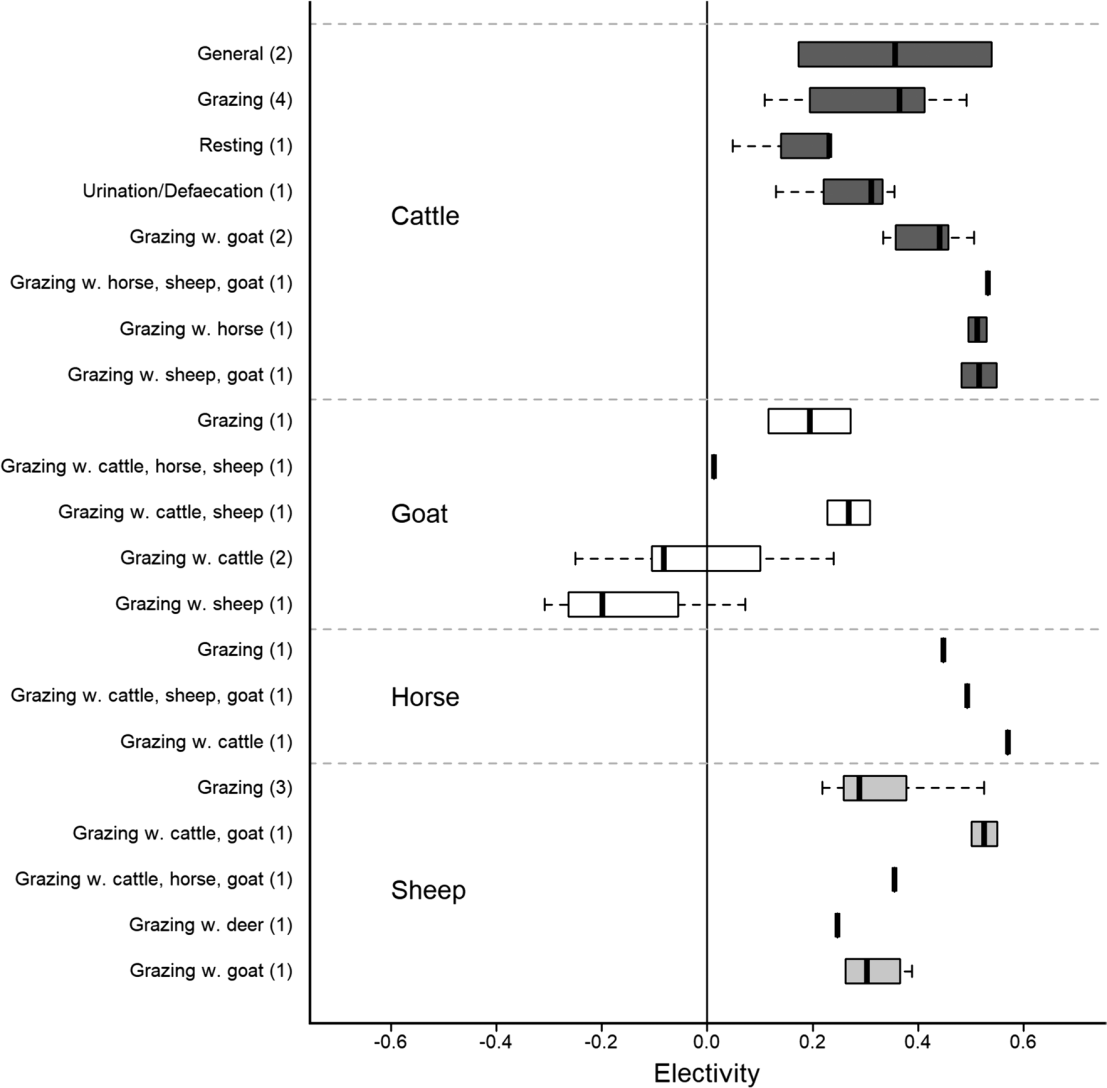
Boxplot of electivity values per grazer species and combination of grazer species. The figure shows electivity for improved areas in (semi-)natural pastures for each animal/animal combination with the measured outcome (general, grazing, resting, urination/defaecation) for each year with data from the included studies. The total number of studies is shown in brackets. Animal combinations are shown as the target species (for which the data relates to) first, and the other species listed after (i.e. if *herd type* = mixed). Electivity values > 0 indicate a preference for the improved area, and values < 0 indicate an avoidance of the improved area (values may in theory range from − 1 to + 1)

Most grazing animals seem to prefer the improved area of the pasture over the semi-natural area. All grazer species in the included studies, except goats, preferred grazing the improved areas regardless of whether they were grazing together with other grazer species or not (Fig. 4). Concerning goats, results from the included studies indicate that they show no preference for the improved areas when grazing together with other grazer species (Additional file 10: Table S1).

**Fig. 5:**
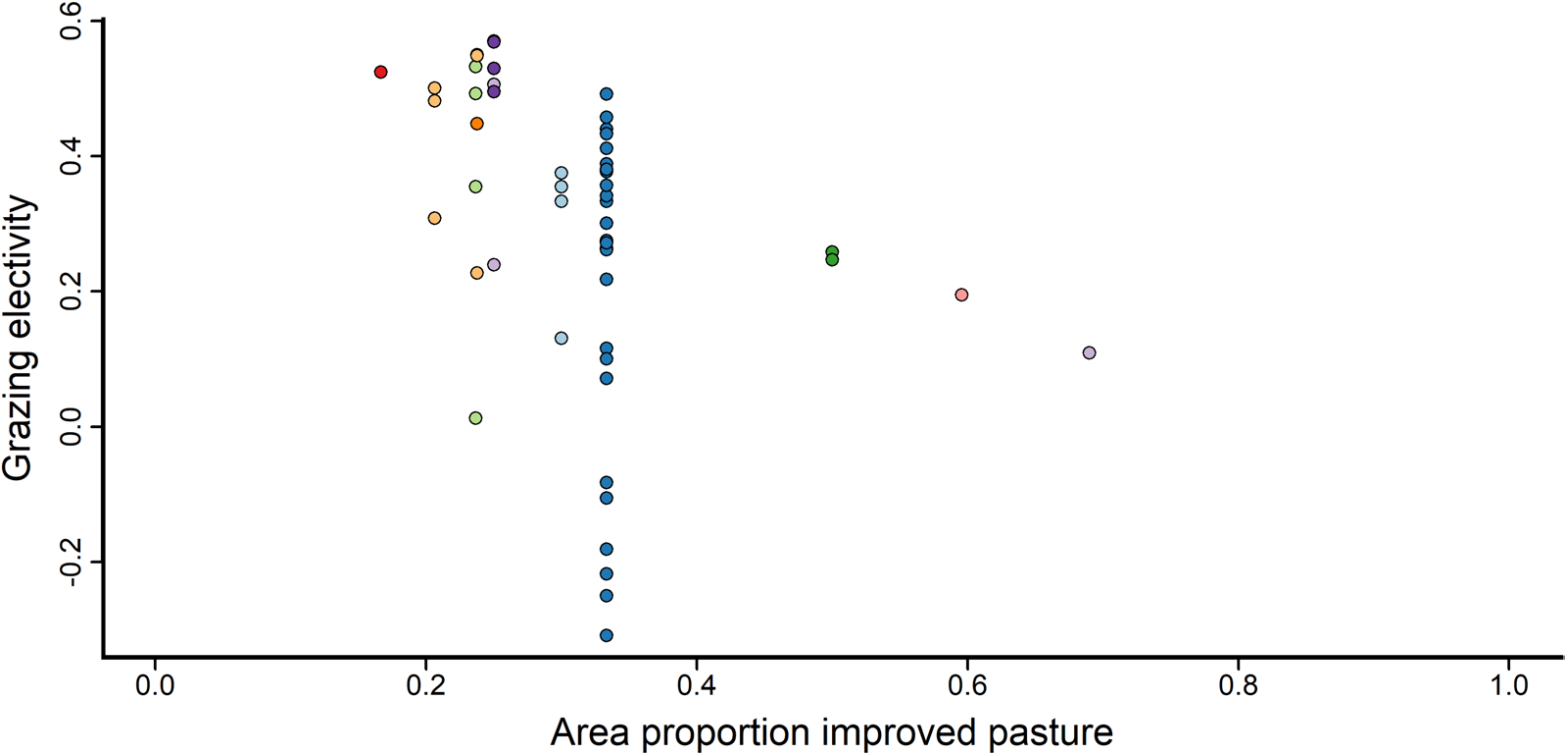
Grazing electivity values in relation to the proportion of improved pasture area. The figure shows the grazers’ electivity for improved areas in (semi-)natural pastures in relation to *proportion of improved pasture* area within the pasture. Electivity values > 0 indicate a preference for the improved area, and values < 0 indicate an avoidance of the improved area (values may in theory range from − 1 to + 1). The figure shows data from all studies included in the analysis, across all *grazer species* and *herd types*. Each study is represented by a unique colour, see Additional file 7 for details

Data from the included studies also indicate that grazing electivity is negatively correlated with the proportional area of the improved pasture area (Fig. 5), indicating that cattle preference for the improved area decreases when the improved area constitutes an increasing proportion of the pasture area, whereas data showed no effect of grazer density (LSU) on electivity (Additional file 10: Table S1). In addition, data indicate that the grazing electivity for improved pasture areas is greater than the resting electivity among cattle, meaning that cattle seem to spend relatively more time grazing than resting in the improved pasture areas (Additional file 10: Table S1).

All patterns concerning electivity measures in relation to improved pasture areas remained statistically significant when excluding data from high risk of bias-studies (Additional file 11: Figs. S1, S2; Additional file 10: Table S2).

Five studies (described in Mosley et al. [51], Clariget et al. [72], Bowman et al. [71], Sowell et al. [76] and Guerrero et al. [73], respectively) contained data on the effects of supplementary feeding on dry matter intake (DMI) of the grazing animals, including three studies on cattle and two on sheep. The limited amount of data restricted the analysis to one overall model, which showed no statistically significant difference in DMI between animals that were fed supplements and those that were not fed supplements (Additional file 11: Fig. S3a; Additional file 10: Table S3). The sensitivity analysis, excluding the high risk of bias studies, did not reveal different results (Additional file 11: Fig. S3b; Additional file 10: Table S4).

## Review limitations

The major limitation of this systematic review was the very small fraction of studies that had research questions, and hence study designs, directly linked to our review questions. Takala et al. [24] was the only study that had a research question and a design that could be directly used to answer Q1. There were more studies with a design that could help answering Q2, but also in these the main study focus was usually on other factors such as productivity, fodder quality and soil chemistry, making it difficult to translate into biodiversity relevance. Most studies that were usable in this review were related to Q3, but very few of them had a study focus on nutrients transport or effects on biodiversity directly. In addition, the included studies targeting Q3 did not present data that were suitable for a standard meta-analysis within the scope of this systematic review. Because of small sample sizes and mismatching study focuses, the risk of overinterpreting our results increases. Therefore, we have tried to be as transparent as possible when analysing the data and presenting our results so as not to mislead the readers.

Another clear limitation was the study designs in the screened literature, from an analytical perspective. Not only were the majority of studies designed to answer another question, but most of them were also less strict concerning replications and controls, both in space and time. This led to the majority of studies being excluded during the full text screening (Fig. 2; Additional file 5).

Important confounding factors, such as study area or animal density, were not consistently reported across the studies. Without these factors, grazing pressure, which is crucial information for understanding the relationship between grazing, soil nutrients and biodiversity, could not be properly accounted for. The differences between study sites, often obvious but difficult to quantify, also limited the ability to draw any general conclusions. The physical environment, i.e., land use structures as well as climatic factors, differed substantially across studies, partly due to the wide spread of studies globally, except for a few clusters of studies from the same research areas (Fig. 3, Additional files 7 and 8).

In addition, our review also included studies with large variations in grazer species. Hence, grazing behaviour and feeding preferences differed between studies, which made interpretation of our quantitative analysis of Q3 difficult. It was therefore not possible to directly translate the results into the conceptual model (Fig. 1). The combination between apparent differences between grazer species and several studies with multiple animal species grazing the same paddock made interpretation even more complex. On the other hand, using several grazing species within the same enclosure is an interesting research design that can both help to disentangle their different and combined effects on biodiversity as well as highlighting potentially more efficient use of semi-natural and natural pastures. However, as this was not the primary question of these studies, it was not possible to do within the scope of our review.

Our choice to perform a broad review, in order to cover as much as possible of the relevant literature targeting our primary review question, had consequences for the review process and potentially also for the conclusions. For example, we did not use any geographical restriction in our eligibility criteria, generating more but potentially less relevant literature. Although most studies were conducted in Europe, many also came from other parts of the world. Transferring knowledge from different types of semi-natural pastures in different parts of the world into policy recommendations for Sweden is not straightforward as many different factors determine responses and relative importance of grazing for biodiversity [12]. Another factor making the review broad, was our use of two supplementary questions to complement the primary review question, which made both the initial work with setting up the review, including search strings, as well as the screening process more time consuming and challenging. In addition, the initial test exercises confirmed our expectations that there would be very few studies targeting our questions specifically and that many studies would be difficult to exclude on abstract level due to abstracts being written very general. To ensure a reliable screening process, we therefore used the inclusive two-step approach for screening titles and abstracts, described in the methods. This means that not all abstracts were double-screened. (However, instead more literature was screened in full text.)

Lastly, for pragmatic reasons we limited our searches to publications written in English, French, Spanish, German, Swedish, Danish and Norwegian. Hence, there may be relevant literature, written in other languages, that we have not been able to find and take into consideration. It is also possible that there is grey literature, which is often harder to find, that was not caught with our search strategy.

## Review conclusions

### Implications for policy/management

Although our literature search gathered a large body of articles, the identified studies could neither be used to answer the primary question (Q1), “What is the effect of giving grazers access to additional nutrient sources on biodiversity in semi-natural pastures?”, nor the supporting question (Q2), “What is the effect of giving grazers access to additional nutrient sources on nutrient status of the soils of semi-natural pastures?”. Some studies were found that were relevant for Q1 and Q2, but they were too few to draw any conclusions on management to support biodiversity in semi-natural pastures. Hence, we stress that further studies specifically designed to answer these questions are needed, particularly to answer our primary question (Q1) (see Implications for research).

Most studies were focused on animal behaviour (Q3), i.e. animal movement, grazing behaviour, intake of fodder through supplementary feeding, and relative grazing time in different pasture types (semi-natural and improved). We found that when animals graze semi-natural pastures that are co-enclosed with improved grassland areas, they tend to prefer to graze the improved pasture. This was statistically significant for cattle, sheep and horses, regardless of whether they grazed together with other grazer species or not. For goats, this effect was statistically significant only when grazing alone, not when grazing together with other grazer species. However, how these types of behaviour would indirectly affect biodiversity and nutrient transport (Fig. 1) is unclear and cannot be directly translated into management recommendations in any direction.

With limited amount of data, we found no statistically significant difference in pasture forage intake between animals that were fed supplements and those that were not.

Based on our findings, we suggest that the current Swedish general requirements and regulations concerning giving the grazers access to additional nutrient sources and its effects on biodiversity in semi-natural pastures should be further discussed in relation to the evidence-base. The risk of adding nutrients, either as supplementary feed or co-enclosure of semi-natural and improved pastures, compared to the risk of semi-natural pastures becoming abandoned as an effect of low profitability [20] should also be elaborated on as a part of Swedish biodiversity protection policy. To acknowledge the potential effect of nutrient addition in semi-natural pastures, broad spatial and temporal scales must be considered in order to capture aspects such as dispersal of organisms and historical management legacies [17, 77].

### Implications for research

In this section, we suggest how research can be designed and conducted to fill the identified knowledge gap and support decision-making.

In principle, designing a study to answer question Q1 is quite straightforward. The lack of such studies is probably more attributed to low interest outside a limited group of practitioners and, in addition, falling between the disciplines of biodiversity and livestock sciences. Although some studies touched upon our Q1, only three studies fulfilled our inclusion criteria. Among these, the study by Takala et al. [24] was the only one clearly addressing our question per se, with a Control-Impact (CI) study on the effects of co-enclosing semi-natural pastures with improved pasture areas.

**Fig. 6:**
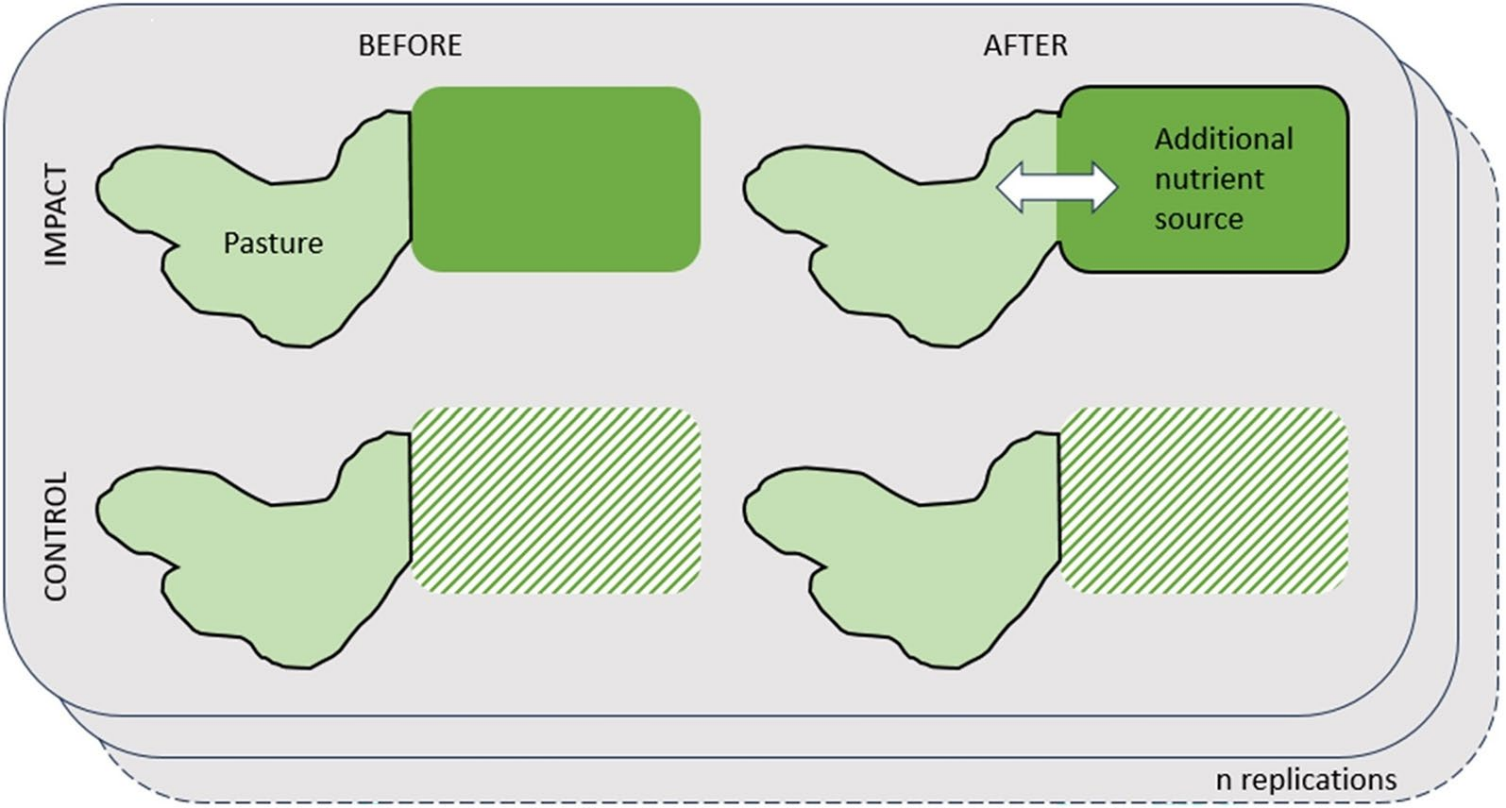
Visualisation of the suggested Before-After-Control-Impact (BACI) study design. The figure shows a suitable study design for evaluating effects on biodiversity in semi-natural pastures of providing an additional nutrient source accessible for the grazing animals (Q1) (either co-enclosing an improved pasture or supplementary feeding; white arrow). Optimally, a study of the impact of co-enclosing an improved pasture has improved pastures also outside the enclosed control pastures (hatched area)

The most suitable design to answer the question is a Before-After-Control-Impact (BACI) design, evaluating the addition of supplementary feeding or co-enclosure with improved pasture areas on biodiversity in the semi-natural pasture (Fig. 6). The BACI design is generally suitable for evaluating biodiversity responses to management interventions [78–81]. Other study designs, such as Before-After (BA) or Control-Impact (CI), are substantially less powerful for determining effect direction and magnitude and more likely to be misleading [78].

The pastures should be assigned to treatment groups (impact or control) in pairs or in blocks along environmental gradients (e.g. regarding wetness, historical management, climate or landscape context). A randomised assignment procedure would be advantageous. To not introduce unintentional bias, all study pastures, including controls, should be selected so that there is potential to include an additional nutrient source (Fig. 6). Biodiversity variables should be evaluated in all pastures before and after the grazers have been given access to the additional nutrient source in the impact group. According to Christie et al. [78], BACI designs identify the direction of the true effect consistently, with reduced uncertainty around the effect size with increasing replication of impact and control sites.

The outcome of nutrient source additions likely depends on a number of management factors and environmental conditions, which may be controlled for by including or excluding the variability of such factors in the study and its analyses. A key aspect to consider in the study design is how to incorporate the grazing pressure in relation to grassland area or productivity. Importantly, the biodiversity outcomes chosen will also affect which effect modifiers that need to be taken into account (Additional file 10: Table S5).

**Fig. 7:**
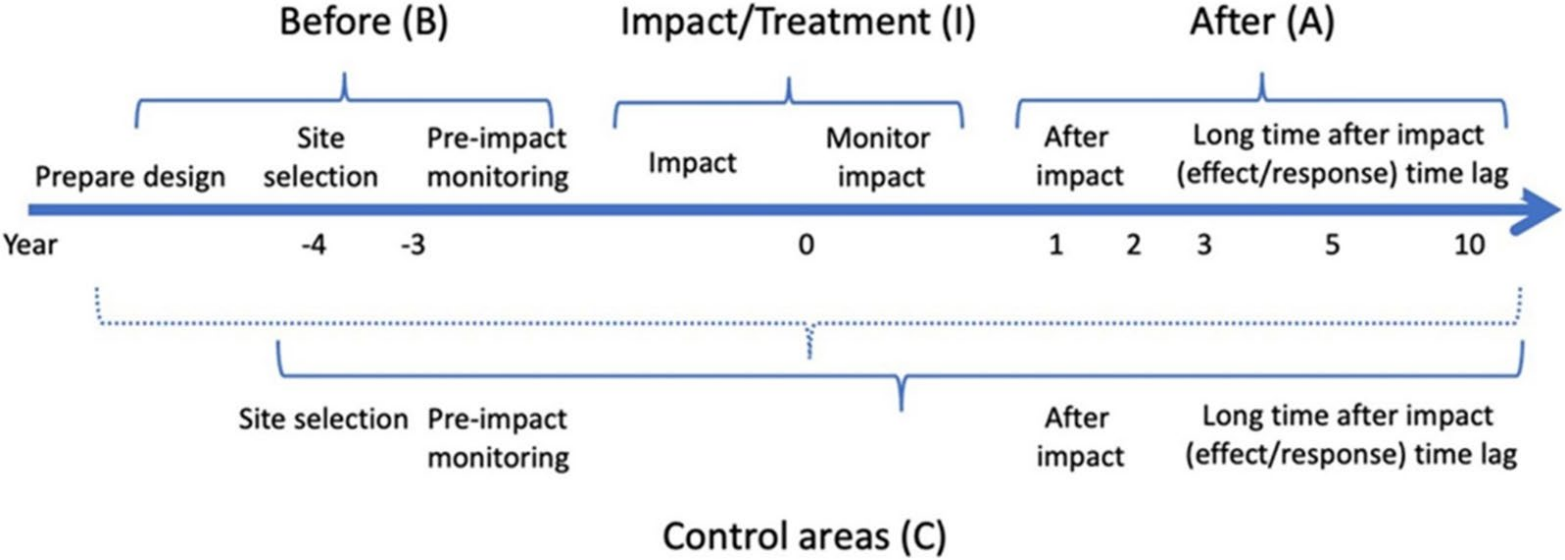
Suggested timeline for a research study of Q1. The figure illustrates the suggested timeline for studying Q1, including pre-impact monitoring of control as well as impact sites, and a sufficient time period after the treatment to capture time-lag effects that may take more than 10 years for organisms with slow dispersal and population dynamics. While biodiversity monitoring should start before or at least immediately after the treatment starts, analytical evaluation of the impact should preferably be made from a few years after the impact/treatment was initiated but should never stop at that time. The “before” period can be substantially shortened if sites and data from ongoing grassland biodiversity monitoring programs are available (see text)

We provide a generalised timeline for planning such a study in Fig. 7. This approach will be especially feasible in countries where background data already exist and can be used to select representative and comparable study sites. In addition, there is an advantage if useful “Before” data on biodiversity also exist, e.g. from national grassland monitoring programs, such as in Sweden [82], the UK [83, 84], Germany [85, 86] and others [86].

## Supplementary Information

Additional file 1: ROSES.

Additional file 2: Search documentation.

Additional file 3: Reviews checked for relevant references.

Additional file 4: Benchmark studies.

Additional file 5: Excluded articles.

Additional file 6: Reasons for validity decisions.

Additional file 7: Database.

Additional file 8: Interactive evidence atlas.

Additional file 9: Unobtainable articles.

Additional file 10: Supplementary tables.

Additional file 11: Supplementary figures.

## Data Availability

All data generated or analysed during this study are included in this published article and its supplementary information files. Intermediate steps of the data extraction (e.g. raw figure data extraction from individual studies) are available upon request.
