# Supplementary material for: Effects on biodiversity in semi-natural pastures of giving the grazing animals access to additional nutrient sources: a systematic review
Source: Environ Evid. 2024 Aug 1;13:18. doi: 10.1186/s13750-024-00343-4 (PMC11378873; doi:10.1186/s13750-024-00343-4)
Supplement: Supplementary file 2 — Additional file 2: Search documentation. [file 13750_2024_343_MOESM2_ESM.docx]

README

Title: Searching for literature

Description: This additional file describes all the search strings that were used to find peer-reviewed articles and grey literature to include in the systematic review. Searches were made in bibliographic databases, an academic search engine and websites of relevant organisations.

**Searching for literature**

**Bibliographic database search**

**Database: Scopus**

Database provider: Elsevier

Date of search: January 18, 2021; September 18, 2023

**Search string for PECO 1 - Outcome: Biodiversity**

| **No** | **Search string** | **Number of hits** (September 18, 2023) |
| --- | --- | --- |
|  | **Population: Semi-natural pastures** |  |
| **1** | (TITLE-ABS-KEY(("semi-natural" OR seminatural OR natural OR "species rich" OR "nutrient poor" OR extensive* OR unimproved OR unfertilized OR alpine* OR mountain* OR forest* OR wood* OR rough OR calcareous OR siliceous OR montan* OR boreal OR temperate OR shore* OR coast* OR wet OR wetted OR flood*) W/5 (pasture* OR pastoral* OR grassland* OR "grass land*" OR graz* OR meadow* OR rangeland* OR "range land*"))) OR (TITLE-ABS-KEY(alvar* OR heath* OR moorland* OR "moor land*" OR dehesa* OR montado* OR "pastoral landscape*")) AND (TITLE-ABS-KEY(graz* OR pasture* OR enclos* OR fenc* OR paddock*)) | **26 649** |
|  | **Exposure: Giving the grazers access to additional nutrient sources** |  |
| **2** | (TITLE-ABS-KEY(ley OR leys OR (("nutrient rich" OR fertilized OR improved OR intensive*) W/3 (pasture* OR pastoral* OR grassland* OR "grass land*")))) OR (TITLE-ABS-KEY((former OR ex OR previous* OR abandon*) W/3 (field* OR farmland* OR "farm land*" OR "arable land*" OR "agricultural land*" OR "cultivated land*" OR cropland* OR "crop land*"))) OR (TITLE-ABS-KEY((heterogen* OR additional OR multi* OR mix* OR divers OR diverse OR adjacent OR neighbour* OR vary* OR varied OR variable OR various OR variation*) W/5 (graz* OR pasture* OR grassland* OR "grass land*" OR enclos* OR fenc* OR paddock* OR habitat* OR biotop*))) OR (TITLE-ABS-KEY("grazing regime*" OR "pasture regime*" OR "pastoral regime*" OR "stocking regime*" OR "grazing management*" OR "pasture management*" OR "pastoral management*" OR "stocking management*" OR "grazing strateg*" OR "pasture strateg*" OR "pastoral strateg*" OR "stocking strateg*" OR "grazing system*" OR "pasture system*" OR "pastoral system*" OR "stocking system*" OR "grazing method*" OR "pasture method*" OR "pastoral method*" OR "stocking method*" OR "grazing practice*" OR "pasture practice*" OR "pastoral practice*" OR "stocking practice*" OR "grazing experiment*" OR "pasture experiment*" OR "pastoral experiment*" OR "stocking experiment*" OR "pasture distribution*" OR "field management*" OR mosaic* OR "same enclosure*" OR ranching OR "ranch farming")) OR (TITLE-ABS-KEY((suppl* OR enrich* OR additional OR creep* OR indoor* OR concentrate*) AND (feed* OR food* OR nutrition* OR fodder* OR forage* OR silage* OR roughage* OR hay* OR mineral*))) | **990 255** |
|  | **Outcome: Biodiversity** |  |
| **3** | (TITLE-ABS-KEY(biodiversity OR "bio-diversity" OR "biological diversity" OR ((species OR insect* OR plant* OR vegetation OR botanic* OR flora* OR floristic* OR fauna* OR faunistic* OR invertebrete* OR vertebrate* OR organism* OR functional OR tax* OR trait*) W/5 (diversity OR abundan* OR richness OR heterogen* OR indicator* OR frequen* OR composition* OR distribution* OR density OR dispers* OR target* OR focal OR keystone OR umbrella OR "red-list*" OR redlist* OR threaten* OR endangered OR rare)))) | **1 394 060** |
|  | **Combination of search strings** |  |
| **4** | 1 AND 2 AND 3 | **3 582** |
|  | **Limit to language: English, Danish, French, German, Norwegian, Spanish or Swedish** |  |
| **5** | AND (LIMIT-TO(LANGUAGE, "English") OR LIMIT-TO(LANGUAGE, "Danish") OR LIMIT-TO(LANGUAGE, "French") OR LIMIT-TO(LANGUAGE, "German") OR LIMIT-TO(LANGUAGE, "Norwegian") OR LIMIT-TO(LANGUAGE, "Spanish") OR LIMIT-TO( LANGUAGE, "Swedish")) | **3 455** |

**Search string for PECO 2 - Outcome: Changed nutrient status of the soils**

| **No** | **Search string** | **Number of hits** (September 18, 2023) |
| --- | --- | --- |
|  | **Population: Semi-natural pastures** |  |
| **1** | (TITLE-ABS-KEY(("semi-natural" OR seminatural OR natural OR "species rich" OR "nutrient poor" OR extensive* OR unimproved OR unfertilized OR alpine* OR mountain* OR forest* OR wood* OR rough OR calcareous OR siliceous OR montan* OR boreal OR temperate OR shore* OR coast* OR wet OR wetted OR flood*) W/5 (pasture* OR pastoral* OR grassland* OR "grass land*" OR graz* OR meadow* OR rangeland* OR "range land*"))) OR (TITLE-ABS-KEY(alvar* OR heath* OR moorland* OR "moor land*" OR dehesa* OR montado* OR "pastoral landscape*")) AND (TITLE-ABS-KEY(graz* OR pasture* OR enclos* OR fenc* OR paddock*)) | **26 649** |
|  | **Exposure: Giving the grazers access to additional nutrient sources** |  |
| **2** | (TITLE-ABS-KEY(ley OR leys OR (("nutrient rich" OR fertilized OR improved OR intensive*) W/3 (pasture* OR pastoral* OR grassland* OR "grass land*")))) OR (TITLE-ABS-KEY((former OR ex OR previous* OR abandon*) W/3 (field* OR farmland* OR "farm land*" OR "arable land*" OR "agricultural land*" OR "cultivated land*" OR cropland* OR "crop land*"))) OR (TITLE-ABS-KEY((heterogen* OR additional OR multi* OR mix* OR divers OR diverse OR adjacent OR neighbour* OR vary* OR varied OR variable OR various OR variation*) W/5 (graz* OR pasture* OR grassland* OR "grass land*" OR enclos* OR fenc* OR paddock* OR habitat* OR biotop*))) OR (TITLE-ABS-KEY("grazing regime*" OR "pasture regime*" OR "pastoral regime*" OR "stocking regime*" OR "grazing management*" OR "pasture management*" OR "pastoral management*" OR "stocking management*" OR "grazing strateg*" OR "pasture strateg*" OR "pastoral strateg*" OR "stocking strateg*" OR "grazing system*" OR "pasture system*" OR "pastoral system*" OR "stocking system*" OR "grazing method*" OR "pasture method*" OR "pastoral method*" OR "stocking method*" OR "grazing practice*" OR "pasture practice*" OR "pastoral practice*" OR "stocking practice*" OR "grazing experiment*" OR "pasture experiment*" OR "pastoral experiment*" OR "stocking experiment*" OR "pasture distribution*" OR "field management*" OR mosaic* OR "same enclosure*" OR ranching OR "ranch farming")) OR (TITLE-ABS-KEY((suppl* OR enrich* OR additional OR creep* OR indoor* OR concentrate*) AND (feed* OR food* OR nutrition* OR fodder* OR forage* OR silage* OR roughage* OR hay* OR mineral*))) | **990 255** |
|  | **Outcome: Changed nutrient status of the soils** |  |
| **3** | (TITLE-ABS-KEY(soil* AND (nutrient* OR "nutritional status" OR "nutritional level*" OR "nutritional value*" OR phosph* OR nitrogen* OR nitrate* OR ammonium* OR potassium* OR calcium* OR magnesium*))) OR (TITLE-ABS-KEY("soil chemistry" OR "soil fertility" OR "soil condition*" OR "soil propert*" OR "plant indicator*" OR ellenberg* OR "biomass production*" OR eutrophi* OR trophic* OR oligotrophi*)) | **556 701** |
|  | **Combination of search strings** |  |
| **4** | 1 AND 2 AND 3 | **1 873** |
|  | **Limit to language: English, Danish, French, German, Norwegian, Spanish or Swedish** |  |
| **5** | AND (LIMIT-TO(LANGUAGE, "English") OR LIMIT-TO(LANGUAGE, "Danish") OR LIMIT-TO(LANGUAGE, "French") OR LIMIT-TO(LANGUAGE, "German") OR LIMIT-TO(LANGUAGE, "Norwegian") OR LIMIT-TO(LANGUAGE, "Spanish") OR LIMIT-TO( LANGUAGE, "Swedish")) | **1 789** |

**Search string for PO - Outcome: Behavioural measures**

| **No** | **Search string** | **Number of hits** (September 18, 2023) |
| --- | --- | --- |
|  | **Population: Grazing domestic animals in semi-natural or natural pastures, that also have access to an additional nutrient source** |  |
| **1** | (TITLE-ABS-KEY(graz* OR herbivor* OR ruminant* OR cattle* OR livestock* OR cow* OR bull OR bulls OR beef OR sheep* OR horse* OR donkey* OR mule* OR goat* OR lama* OR llama* OR alpaca*)) AND ((TITLE-ABS-KEY(("semi-natural" OR seminatural OR natural OR "species rich" OR "nutrient poor" OR extensive* OR unimproved OR unfertilized OR alpine* OR mountain* OR forest* OR wood* OR rough OR calcareous OR siliceous OR montan* OR boreal OR temperate OR shore* OR coast* OR wet OR wetted OR flood*) W/5 (pasture* OR pastoral* OR grassland* OR "grass land*" OR graz* OR meadow* OR rangeland* OR "range land*"))) OR (TITLE-ABS-KEY(alvar* OR heath* OR moorland* OR "moor land*" OR dehesa* OR montado* OR "pastoral landscape*")) AND (TITLE-ABS-KEY(graz* OR pasture* OR enclos* OR fenc* OR paddock*))) AND ((TITLE-ABS-KEY(ley OR leys OR (("nutrient rich" OR fertilized OR improved OR intensive*) W/3 (pasture* OR pastoral* OR grassland* OR "grass land*")))) OR (TITLE-ABS-KEY((former OR ex OR previous* OR abandon*) W/3 (field* OR farmland* OR "farm land*" OR "arable land*" OR "agricultural land*" OR "cultivated land*" OR cropland* OR "crop land*"))) OR (TITLE-ABS-KEY((heterogen* OR additional OR multi* OR mix* OR divers OR diverse OR adjacent OR neighbour* OR vary* OR varied OR variable OR various OR variation*) W/5 (graz* OR pasture* OR grassland* OR "grass land*" OR enclos* OR fenc* OR paddock* OR habitat* OR biotop*))) OR (TITLE-ABS-KEY("grazing regime*" OR "pasture regime*" OR "pastoral regime*" OR "stocking regime*" OR "grazing management*" OR "pasture management*" OR "pastoral management*" OR "stocking management*" OR "grazing strateg*" OR "pasture strateg*" OR "pastoral strateg*" OR "stocking strateg*" OR "grazing system*" OR "pasture system*" OR "pastoral system*" OR "stocking system*" OR "grazing method*" OR "pasture method*" OR "pastoral method*" OR "stocking method*" OR "grazing practice*" OR "pasture practice*" OR "pastoral practice*" OR "stocking practice*" OR "grazing experiment*" OR "pasture experiment*" OR "pastoral experiment*" OR "stocking experiment*" OR "pasture distribution*" OR "field management*" OR mosaic* OR "same enclosure*" OR ranching OR "ranch farming")) OR (TITLE-ABS-KEY((suppl* OR enrich* OR additional OR creep* OR indoor* OR concentrate*) AND (feed* OR food* OR nutrition* OR fodder* OR forage* OR silage* OR roughage* OR hay* OR mineral*)))) | **7 335** |
|  | **Outcome: Behavioural measures** |  |
| **2** | (TITLE-ABS-KEY(behaviour* OR behavior* OR move* OR moving OR trampl* OR tread* OR scrape OR scraping OR urin* OR excret* OR defecat* OR feces OR faeces OR dung* OR manure* OR fouling OR "stock* distribution*" OR "livestock distribution*" OR "herd distribution*" OR "grazing distribution*" OR "grazing pressure*" OR "grazing pattern*" OR "grazing time*" OR "grazing period*" OR "grazing intensit*" OR "grazing habit" OR "grazing habits" OR "under-graz*" OR undergraz* OR "over-graz*" OR overgraz* OR foraging OR "resting time*" OR "resting period*")) OR (TITLE-ABS-KEY((graz* OR diet* OR eat* OR feed* OR food* OR fodder* OR forag*) W/5 (select* OR prefer* OR choose OR choice))) | **9 956 853** |
|  | **Combination of search strings** |  |
| **3** | 1 AND 2 | **2 957** |
|  | **Limit to language: English, Danish, French, German, Norwegian, Spanish or Swedish** |  |
| **4** | AND (LIMIT-TO(LANGUAGE, "English") OR LIMIT-TO(LANGUAGE, "Danish") OR LIMIT-TO(LANGUAGE, "French") OR LIMIT-TO(LANGUAGE, "German") OR LIMIT-TO(LANGUAGE, "Norwegian") OR LIMIT-TO(LANGUAGE, "Spanish") OR LIMIT-TO( LANGUAGE, "Swedish")) | **2 831** |

* = Represents any group of characters, including no character

" " = Searches for an exact phrase

TITLE-ABS-KEY = Title or Abstract or Keywords

W/5 = A proximity operator to find terms within five words from each other

W/3 = A proximity operator to find terms within three words from each other

**Database: Web of Science Core Collection (1970-)**

Database provider: Clarivate Analytics

Date of search: January 18, 2021; September 18, 2023

Including: Science Citation Index Expanded (SCI-EXPANDED), Social Sciences Citation Index (SSCI), Arts & Humanities Citation Index (A&HCI), Conference Proceedings Citation Index- Science (CPCI-S), Conference Proceedings Citation Index- Social Science & Humanities (CPCI-SSH) and Emerging Sources Citation Index (ESCI)

**Search string for PECO 1 - Outcome: Biodiversity**

| **No** | **Search string** | **Number of hits** (September 18, 2023) |
| --- | --- | --- |
|  | **Population: Semi-natural pastures** |  |
| **1** | TS=((("semi-natural" OR seminatural OR natural OR "species rich" OR "nutrient poor" OR extensive* OR unimproved OR unfertilized OR alpine* OR mountain* OR forest* OR wood* OR rough OR calcareous OR siliceous OR montan* OR boreal OR temperate OR shore* OR coast* OR wet OR wetted OR flood*) NEAR/5 (pasture* OR pastoral* OR grassland* OR "grass land*" OR graz* OR meadow* OR rangeland* OR "range land*")) OR (alvar* OR heath* OR moorland* OR "moor land*" OR dehesa* OR montado* OR "pastoral landscape*")) AND TS=(graz* OR pasture* OR enclos* OR fenc* OR paddock*) | **22 406** |
|  | **Exposure: Giving the grazers access to additional nutrient sources** |  |
| **2** | TS=(ley OR leys OR (("nutrient rich" OR fertilized OR improved OR intensive*) NEAR/3 (pasture* OR pastoral* OR grassland* OR "grass land*"))) OR TS=((former OR ex OR previous* OR abandon*) NEAR/3 (field* OR farmland* OR "farm land*" OR "arable land*" OR "agricultural land*" OR "cultivated land*" OR cropland* OR "crop land*")) OR TS=((heterogen* OR additional OR multi* OR mix* OR divers OR diverse OR adjacent OR neighbour* OR vary* OR varied OR variable OR various OR variation*) NEAR/5 (graz* OR pasture* OR grassland* OR "grass land*" OR enclos* OR fenc* OR paddock* OR habitat* OR biotop*)) OR TS=("grazing regime*" OR "pasture regime*" OR "pastoral regime*" OR "stocking regime*" OR "grazing management*" OR "pasture management*" OR "pastoral management*" OR "stocking management*" OR "grazing strateg*" OR "pasture strateg*" OR "pastoral strateg*" OR "stocking strateg*" OR "grazing system*" OR "pasture system*" OR "pastoral system*" OR "stocking system*" OR "grazing method*" OR "pasture method*" OR "pastoral method*" OR "stocking method*" OR "grazing practice*" OR "pasture practice*" OR "pastoral practice*" OR "stocking practice*" OR "grazing experiment*" OR "pasture experiment*" OR "pastoral experiment*" OR "stocking experiment*" OR "pasture distribution*" OR "field management*" OR mosaic* OR "same enclosure*" OR ranching OR "ranch farming") OR TS=((suppl* OR enrich* OR additional OR creep* OR indoor* OR concentrate*) AND (feed* OR food* OR nutrition* OR fodder* OR forage* OR silage* OR roughage* OR hay* OR mineral*)) | **730 477** |
|  | **Outcome: Biodiversity** |  |
| **3** | TS=(biodiversity OR "bio-diversity" OR "biological diversity" OR ((species OR insect* OR plant* OR vegetation OR botanic* OR flora* OR floristic* OR fauna* OR faunistic* OR invertebrete* OR vertebrate* OR organism* OR functional OR tax* OR trait*) NEAR/5 (diversity OR abundan* OR richness OR heterogen* OR indicator* OR frequen* OR composition* OR distribution* OR density OR dispers* OR target* OR focal OR keystone OR umbrella OR "red-list*" OR redlist* OR threaten* OR endangered OR rare))) | **1 150 612** |
|  | **Combination of search strings** |  |
| **4** | 1 AND 2 AND 3 | **3 579** |
|  | **Limit to language: English, Danish, French, German, Norwegian, Spanish or Swedish** |  |
| **5** | AND LANGUAGE: (English OR Danish OR French OR German OR Norwegian OR Spanish OR Swedish) | **3 532** |

**Search string for PECO 2 - Outcome: Changed nutrient status of the soils**

| **No** | **Search string** | **Number of hits** (September 18, 2023) |
| --- | --- | --- |
|  | **Population: Semi-natural pastures** |  |
| **1** | TS=((("semi-natural" OR seminatural OR natural OR "species rich" OR "nutrient poor" OR extensive* OR unimproved OR unfertilized OR alpine* OR mountain* OR forest* OR wood* OR rough OR calcareous OR siliceous OR montan* OR boreal OR temperate OR shore* OR coast* OR wet OR wetted OR flood*) NEAR/5 (pasture* OR pastoral* OR grassland* OR "grass land*" OR graz* OR meadow* OR rangeland* OR "range land*")) OR (alvar* OR heath* OR moorland* OR "moor land*" OR dehesa* OR montado* OR "pastoral landscape*")) AND TS=(graz* OR pasture* OR enclos* OR fenc* OR paddock*) | **22 407** |
|  | **Exposure: Giving the grazers access to additional nutrient sources** |  |
| **2** | TS=(ley OR leys OR (("nutrient rich" OR fertilized OR improved OR intensive*) NEAR/3 (pasture* OR pastoral* OR grassland* OR "grass land*"))) OR TS=((former OR ex OR previous* OR abandon*) NEAR/3 (field* OR farmland* OR "farm land*" OR "arable land*" OR "agricultural land*" OR "cultivated land*" OR cropland* OR "crop land*")) OR TS=((heterogen* OR additional OR multi* OR mix* OR divers OR diverse OR adjacent OR neighbour* OR vary* OR varied OR variable OR various OR variation*) NEAR/5 (graz* OR pasture* OR grassland* OR "grass land*" OR enclos* OR fenc* OR paddock* OR habitat* OR biotop*)) OR TS=("grazing regime*" OR "pasture regime*" OR "pastoral regime*" OR "stocking regime*" OR "grazing management*" OR "pasture management*" OR "pastoral management*" OR "stocking management*" OR "grazing strateg*" OR "pasture strateg*" OR "pastoral strateg*" OR "stocking strateg*" OR "grazing system*" OR "pasture system*" OR "pastoral system*" OR "stocking system*" OR "grazing method*" OR "pasture method*" OR "pastoral method*" OR "stocking method*" OR "grazing practice*" OR "pasture practice*" OR "pastoral practice*" OR "stocking practice*" OR "grazing experiment*" OR "pasture experiment*" OR "pastoral experiment*" OR "stocking experiment*" OR "pasture distribution*" OR "field management*" OR mosaic* OR "same enclosure*" OR ranching OR "ranch farming") OR TS=((suppl* OR enrich* OR additional OR creep* OR indoor* OR concentrate*) AND (feed* OR food* OR nutrition* OR fodder* OR forage* OR silage* OR roughage* OR hay* OR mineral*)) | **730 511** |
|  | **Outcome: Changed nutrient status of the soils** |  |
| **3** | TS=(soil* AND (nutrient* OR "nutritional status" OR "nutritional level*" OR "nutritional value*" OR phosph* OR nitrogen* OR nitrate* OR ammonium* OR potassium* OR calcium* OR magnesium*)) OR TS=("soil chemistry" OR "soil fertility" OR "soil condition*" OR "soil propert*" OR "plant indicator*" OR ellenberg* OR "biomass production*" OR eutrophi* OR trophic* OR oligotrophi*) | **458 341** |
|  | **Combination of search strings** |  |
| **4** | 1 AND 2 AND 3 | **2 009** |
|  | **Limit to language: English, Danish, French, German, Norwegian, Spanish or Swedish** |  |
| **5** | AND LANGUAGE: (English OR Danish OR French OR German OR Norwegian OR Spanish OR Swedish) | **1 970** |

**Search string for PO - Outcome: Behavioural measures**

| **No** | **Search string** | **Number of hits** (September 18, 2023) |
| --- | --- | --- |
|  | **Population: Grazing domestic animals in semi-natural or natural pastures, that also have access to an additional nutrient source** |  |
| **1** | TS=(graz* OR herbivor* OR ruminant* OR cattle* OR livestock* OR cow* OR bull OR bulls OR beef OR sheep* OR horse* OR donkey* OR mule* OR goat* OR lama* OR llama* OR alpaca*) AND TS=((("semi-natural" OR seminatural OR natural OR "species rich" OR "nutrient poor" OR extensive* OR unimproved OR unfertilized OR alpine* OR mountain* OR forest* OR wood* OR rough OR calcareous OR siliceous OR montan* OR boreal OR temperate OR shore* OR coast* OR wet OR wetted OR flood*) NEAR/5 (pasture* OR pastoral* OR grassland* OR "grass land*" OR graz* OR meadow* OR rangeland* OR "range land*")) OR (alvar* OR heath* OR moorland* OR "moor land*" OR dehesa* OR montado* OR "pastoral landscape*")) AND TS=(graz* OR pasture* OR enclos* OR fenc* OR paddock*) AND (TS=(ley OR leys OR (("nutrient rich" OR fertilized OR improved OR intensive*) NEAR/3 (pasture* OR pastoral* OR grassland* OR "grass land*"))) OR TS=((former OR ex OR previous* OR abandon*) NEAR/3 (field* OR farmland* OR "farm land*" OR "arable land*" OR "agricultural land*" OR "cultivated land*" OR cropland* OR "crop land*")) OR TS=((heterogen* OR additional OR multi* OR mix* OR divers OR diverse OR adjacent OR neighbour* OR vary* OR varied OR variable OR various OR variation*) NEAR/5 (graz* OR pasture* OR grassland* OR "grass land*" OR enclos* OR fenc* OR paddock* OR habitat* OR biotop*)) OR TS=("grazing regime*" OR "pasture regime*" OR "pastoral regime*" OR "stocking regime*" OR "grazing management*" OR "pasture management*" OR "pastoral management*" OR "stocking management*" OR "grazing strateg*" OR "pasture strateg*" OR "pastoral strateg*" OR "stocking strateg*" OR "grazing system*" OR "pasture system*" OR "pastoral system*" OR "stocking system*" OR "grazing method*" OR "pasture method*" OR "pastoral method*" OR "stocking method*" OR "grazing practice*" OR "pasture practice*" OR "pastoral practice*" OR "stocking practice*" OR "grazing experiment*" OR "pasture experiment*" OR "pastoral experiment*" OR "stocking experiment*" OR "pasture distribution*" OR "field management*" OR mosaic* OR "same enclosure*" OR ranching OR "ranch farming") OR TS=((suppl* OR enrich* OR additional OR creep* OR indoor* OR concentrate*) AND (feed* OR food* OR nutrition* OR fodder* OR forage* OR silage* OR roughage* OR hay* OR mineral*))) | **6 672** |
|  | **Outcome: Behavioural measures** |  |
| **2** | TS=(behaviour* OR behavior* OR move* OR moving OR trampl* OR tread* OR scrape OR scraping OR urin* OR excret* OR defecat* OR feces OR faeces OR dung* OR manure* OR fouling OR "stock* distribution*" OR "livestock distribution*" OR "herd distribution*" OR "grazing distribution*" OR "grazing pressure*" OR "grazing pattern*" OR "grazing time*" OR "grazing period*" OR "grazing intensit*" OR "grazing habit" OR "grazing habits" OR "under-graz*" OR undergraz* OR "over-graz*" OR overgraz* OR foraging OR "resting time*" OR "resting period*") OR TS=((graz* OR diet* OR eat* OR feed* OR food* OR fodder* OR forag*) NEAR/5 (select* OR prefer* OR choose OR choice)) | **7 333 085** |
|  | **Combination of search strings** |  |
| **3** | 1 AND 2 | **3 414** |
|  | **Limit to language: English, Danish, French, German, Norwegian, Spanish or Swedish** |  |
| **4** | AND LANGUAGE: (English OR Danish OR French OR German OR Norwegian OR Spanish OR Swedish) | **3 338** |

* = Represents any group of characters, including no character

" " = Searches for an exact phrase

TS = Topic Search (search the Title, Abstract, Author Keywords and Keywords Plus within every record)

NEAR/5 = A proximity operator to find terms within five words from each other

NEAR/3 = A proximity operator to find terms within three words from each other

**Database: CAB Abstracts (1973-)**

Database provider: Ovid

Date of search: January 19, 2021; September 18, 2023

**Search string for PECO 1 - Outcome: Biodiversity**

| **No** | **Search string** | **Number of hits** (September 18, 2023) |
| --- | --- | --- |
|  | **Population: Semi-natural pastures** |  |
| **1** | (((("semi-natural" OR seminatural OR natural OR "species rich" OR "nutrient poor" OR extensive* OR unimproved OR unfertilized OR alpine* OR mountain* OR forest* OR wood* OR rough OR calcareous OR siliceous OR montan* OR boreal OR temperate OR shore* OR coast* OR wet OR wetted OR flood*) ADJ5 (pasture* OR pastoral* OR grassland* OR "grass land*" OR graz* OR meadow* OR rangeland* OR "range land*")) OR (alvar* OR heath* OR moorland* OR "moor land*" OR dehesa* OR montado* OR "pastoral landscape*")) AND (graz* OR pasture* OR enclos* OR fenc* OR paddock*)).ti,ab,hw. | **42 340** |
|  | **Exposure: Giving the grazers access to additional nutrient sources** |  |
| **2** | (ley OR leys OR (("nutrient rich" OR fertilized OR improved OR intensive*) ADJ3 (pasture* OR pastoral* OR grassland* OR "grass land*")) OR ((former OR ex OR previous* OR abandon*) ADJ3 (field* OR farmland* OR "farm land*" OR "arable land*" OR "agricultural land*" OR "cultivated land*" OR cropland* OR "crop land*")) OR ((heterogen* OR additional OR multi* OR mix* OR divers OR diverse OR adjacent OR neighbour* OR vary* OR varied OR variable OR various OR variation*) ADJ5 (graz* OR pasture* OR grassland* OR "grass land*" OR enclos* OR fenc* OR paddock* OR habitat* OR biotop*)) OR ("grazing regime*" OR "pasture regime*" OR "pastoral regime*" OR "stocking regime*" OR "grazing management*" OR "pasture management*" OR "pastoral management*" OR "stocking management*" OR "grazing strateg*" OR "pasture strateg*" OR "pastoral strateg*" OR "stocking strateg*" OR "grazing system*" OR "pasture system*" OR "pastoral system*" OR "stocking system*" OR "grazing method*" OR "pasture method*" OR "pastoral method*" OR "stocking method*" OR "grazing practice*" OR "pasture practice*" OR "pastoral practice*" OR "stocking practice*" OR "grazing experiment*" OR "pasture experiment*" OR "pastoral experiment*" OR "stocking experiment*" OR "pasture distribution*" OR "field management*" OR mosaic* OR "same enclosure*" OR ranching OR "ranch farming") OR ((suppl* OR enrich* OR additional OR creep* OR indoor* OR concentrate*) AND (feed* OR food* OR nutrition* OR fodder* OR forage* OR silage* OR roughage* OR hay* OR mineral*))).ti,ab,hw. | **647 988** |
|  | **Outcome: Biodiversity** |  |
| **3** | (biodiversity OR "bio-diversity" OR "biological diversity" OR ((species OR insect* OR plant* OR vegetation OR botanic* OR flora* OR floristic* OR fauna* OR faunistic* OR invertebrete* OR vertebrate* OR organism* OR functional OR tax* OR trait*) ADJ5 (diversity OR abundan* OR richness OR heterogen* OR indicator* OR frequen* OR composition* OR distribution* OR density OR dispers* OR target* OR focal OR keystone OR umbrella OR "red-list*" OR redlist* OR threaten* OR endangered OR rare))).ti,ab,hw. | **1 027 961** |
|  | **Combination of search strings** |  |
| **4** | 1 AND 2 AND 3 | **5 618** |
|  | **Limit to language: English, Danish, French, German, Norwegian, Spanish or Swedish** |  |
| **5** | Limit 4 to (english OR danish OR french OR german OR norwegian OR spanish OR swedish) | **5 200** |

**Search string for PECO 2 - Outcome: Changed nutrient status of the soils**

| **No** | **Search string** | **Number of hits** (September 18, 2023) |
| --- | --- | --- |
|  | **Population: Semi-natural pastures** |  |
| **1** | (((("semi-natural" OR seminatural OR natural OR "species rich" OR "nutrient poor" OR extensive* OR unimproved OR unfertilized OR alpine* OR mountain* OR forest* OR wood* OR rough OR calcareous OR siliceous OR montan* OR boreal OR temperate OR shore* OR coast* OR wet OR wetted OR flood*) ADJ5 (pasture* OR pastoral* OR grassland* OR "grass land*" OR graz* OR meadow* OR rangeland* OR "range land*")) OR (alvar* OR heath* OR moorland* OR "moor land*" OR dehesa* OR montado* OR "pastoral landscape*")) AND (graz* OR pasture* OR enclos* OR fenc* OR paddock*)).ti,ab,hw. | **42 340** |
|  | **Exposure: Giving the grazers access to additional nutrient sources** |  |
| **2** | (ley OR leys OR (("nutrient rich" OR fertilized OR improved OR intensive*) ADJ3 (pasture* OR pastoral* OR grassland* OR "grass land*")) OR ((former OR ex OR previous* OR abandon*) ADJ3 (field* OR farmland* OR "farm land*" OR "arable land*" OR "agricultural land*" OR "cultivated land*" OR cropland* OR "crop land*")) OR ((heterogen* OR additional OR multi* OR mix* OR divers OR diverse OR adjacent OR neighbour* OR vary* OR varied OR variable OR various OR variation*) ADJ5 (graz* OR pasture* OR grassland* OR "grass land*" OR enclos* OR fenc* OR paddock* OR habitat* OR biotop*)) OR ("grazing regime*" OR "pasture regime*" OR "pastoral regime*" OR "stocking regime*" OR "grazing management*" OR "pasture management*" OR "pastoral management*" OR "stocking management*" OR "grazing strateg*" OR "pasture strateg*" OR "pastoral strateg*" OR "stocking strateg*" OR "grazing system*" OR "pasture system*" OR "pastoral system*" OR "stocking system*" OR "grazing method*" OR "pasture method*" OR "pastoral method*" OR "stocking method*" OR "grazing practice*" OR "pasture practice*" OR "pastoral practice*" OR "stocking practice*" OR "grazing experiment*" OR "pasture experiment*" OR "pastoral experiment*" OR "stocking experiment*" OR "pasture distribution*" OR "field management*" OR mosaic* OR "same enclosure*" OR ranching OR "ranch farming") OR ((suppl* OR enrich* OR additional OR creep* OR indoor* OR concentrate*) AND (feed* OR food* OR nutrition* OR fodder* OR forage* OR silage* OR roughage* OR hay* OR mineral*))).ti,ab,hw. | **647 988** |
|  | **Outcome: Changed nutrient status of the soils** |  |
| **3** | ((soil* AND (nutrient* OR "nutritional status" OR "nutritional level*" OR "nutritional value*" OR phosph* OR nitrogen* OR nitrate* OR ammonium* OR potassium* OR calcium* OR magnesium*)) OR ("soil chemistry" OR "soil fertility" OR "soil condition*" OR "soil propert*" OR "plant indicator*" OR ellenberg* OR "biomass production*" OR eutrophi* OR trophic* OR oligotrophi*)).ti,ab,hw. | **560 378** |
|  | **Combination of search strings** |  |
| **4** | 1 AND 2 AND 3 | **3 187** |
|  | **Limit to language: English, Danish, French, German, Norwegian, Spanish or Swedish** |  |
| **5** | Limit 4 to (english OR danish OR french OR german OR norwegian OR spanish OR swedish) | **2 903** |

**Search string for PO - Outcome: Behavioural measures**

| **No** | **Search string** | **Number of hits** (September 18, 2023) |
| --- | --- | --- |
|  | **Population: Grazing domestic animals in semi-natural or natural pastures, that also have access to an additional nutrient source** |  |
| **1** | ((graz* OR herbivor* OR ruminant* OR cattle* OR livestock* OR cow* OR bull OR bulls OR beef OR sheep* OR horse* OR donkey* OR mule* OR goat* OR lama* OR llama* OR alpaca*) AND (((("semi-natural" OR seminatural OR natural OR "species rich" OR "nutrient poor" OR extensive* OR unimproved OR unfertilized OR alpine* OR mountain* OR forest* OR wood* OR rough OR calcareous OR siliceous OR montan* OR boreal OR temperate OR shore* OR coast* OR wet OR wetted OR flood*) ADJ5 (pasture* OR pastoral* OR grassland* OR "grass land*" OR graz* OR meadow* OR rangeland* OR "range land*")) OR (alvar* OR heath* OR moorland* OR "moor land*" OR dehesa* OR montado* OR "pastoral landscape*")) AND (graz* OR pasture* OR enclos* OR fenc* OR paddock*) AND (ley OR leys OR (("nutrient rich" OR fertilized OR improved OR intensive*) ADJ3 (pasture* OR pastoral* OR grassland* OR "grass land*")) OR ((former OR ex OR previous* OR abandon*) ADJ3 (field* OR farmland* OR "farm land*" OR "arable land*" OR "agricultural land*" OR "cultivated land*" OR cropland* OR "crop land*")) OR ((heterogen* OR additional OR multi* OR mix* OR divers OR diverse OR adjacent OR neighbour* OR vary* OR varied OR variable OR various OR variation*) ADJ5 (graz* OR pasture* OR grassland* OR "grass land*" OR enclos* OR fenc* OR paddock* OR habitat* OR biotop*)) OR ("grazing regime*" OR "pasture regime*" OR "pastoral regime*" OR "stocking regime*" OR "grazing management*" OR "pasture management*" OR "pastoral management*" OR "stocking management*" OR "grazing strateg*" OR "pasture strateg*" OR "pastoral strateg*" OR "stocking strateg*" OR "grazing system*" OR "pasture system*" OR "pastoral system*" OR "stocking system*" OR "grazing method*" OR "pasture method*" OR "pastoral method*" OR "stocking method*" OR "grazing practice*" OR "pasture practice*" OR "pastoral practice*" OR "stocking practice*" OR "grazing experiment*" OR "pasture experiment*" OR "pastoral experiment*" OR "stocking experiment*" OR "pasture distribution*" OR "field management*" OR mosaic* OR "same enclosure*" OR ranching OR "ranch farming") OR ((suppl* OR enrich* OR additional OR creep* OR indoor* OR concentrate*) AND (feed* OR food* OR nutrition* OR fodder* OR forage* OR silage* OR roughage* OR hay* OR mineral*))))).ti,ab,hw. | **10 272** |
|  | **Outcome: Behavioural measures** |  |
| **2** | (behaviour* OR behavior* OR move* OR moving OR trampl* OR tread* OR scrape OR scraping OR urin* OR excret* OR defecat* OR feces OR faeces OR dung* OR manure* OR fouling OR "stock* distribution*" OR "livestock distribution*" OR "herd distribution*" OR "grazing distribution*" OR "grazing pressure*" OR "grazing pattern*" OR "grazing time*" OR "grazing period*" OR "grazing intensit*" OR "grazing habit" OR "grazing habits" OR "under-graz*" OR undergraz* OR "over-graz*" OR overgraz* OR foraging OR "resting time*" OR "resting period*") OR ((graz* OR diet* OR eat* OR feed* OR food* OR fodder* OR forag*) ADJ5 (select* OR prefer* OR choose OR choice)).ti,ab,hw. | **1 012 707** |
|  | **Combination of search strings** |  |
| **3** | 1 AND 2 | **3 439** |
|  | **Limit to language: English, Danish, French, German, Norwegian, Spanish or Swedish** |  |
| **4** | Limit 3 to (english OR danish OR french OR german OR norwegian OR spanish OR swedish) | **3 150** |

* = Represents any group of characters, including no character
" " = Searches for an exact phrase
.ti,ab,hw. = Title or Abstract or Heading words

ADJ5 = A proximity operator to find terms within five words from each other

ADJ3 = A proximity operator to find terms within three words from each other

**Database: Directory of Open Access Journals (DOAJ)**

Database provider: Independent
Date of search: January 20, 2021

DOAJ is a community-curated list of open access journals and aims to be the starting point for information searches for quality, peer reviewed open access material.

| **Search string** (search in all fields) | **Number of hits** (January 20, 2021) |
| --- | --- |
| **PECO 1 - Outcome: Biodiversity**  "semi-natural" AND pasture* AND biodiversity  "semi-natural" AND grassland* AND biodiversity  "semi-natural" AND "grass land*" AND biodiversity  "semi-natural" AND pasture* AND "biological diversity"  "semi-natural" AND grassland* AND "biological diversity"  "semi-natural" AND "grass land*" AND "biological diversity"  **PECO 2 - Outcome: Changed nutrient status of the soils**  "semi-natural" AND pasture* AND nutrient*  "semi-natural" AND grassland* AND nutrient*  "semi-natural" AND "grass land*" AND nutrient*  **PO - Outcome: Behavioural measures**  "semi-natural" AND pasture* AND behav*  "semi-natural" AND grassland* AND behav*  "semi-natural" AND "grass land*" AND behav*  "semi-natural" AND pasture* AND mov*  "semi-natural" AND grassland* AND mov*  "semi-natural" AND "grass land*" AND mov* | **23**  **63**  **0**  **1**  **3**  **0**  **6**  **12**  **0**  **3**  **2**  **0**  **2**  **1**  **0** |

* = Represents any group of characters, including no character
" " = Searches for an exact phrase

**Database: DiVA**

Database provider: Swedish universities and research institutions
Date of search: January 20, 2021; September 19, 2023

DiVA contains research publications and student theses from Swedish universities and research institutions.

| **Language** | **Search string** (search in all fields) | **Number of hits** (September 19, 2023) |
| --- | --- | --- |
| **English** | **PECO 1 - Outcome: Biodiversity**  "semi-natural" AND pasture* AND biodiversity  "semi-natural" AND grassland* AND biodiversity  "semi-natural" AND "grass land*" AND biodiversity  "semi-natural" AND pasture* AND "biological diversity"  "semi-natural" AND grassland* AND "biological diversity"  "semi-natural" AND "grass land*" AND "biological diversity"  **PECO 2 - Outcome: Changed nutrient status of the soils**  "semi-natural" AND pasture* AND nutrient*  "semi-natural" AND grassland* AND nutrient*  "semi-natural" AND "grass land*" AND nutrient*  **PO - Outcome: Behavioural measures**  "semi-natural" AND pasture* AND behav*  "semi-natural" AND grassland* AND behav*  "semi-natural" AND "grass land*" AND behav*  "semi-natural" AND pasture* AND mov*  "semi-natural" AND grassland* AND mov*  "semi-natural" AND "grass land*" AND mov* | **26**  **97**  **0**  **3**  **9**  **0**  **6**  **13**  **0**  **0**  **1**  **0**  **3**  **3**  **0** |
| **Swedish** | **PECO 1 - Outcome: Biodiversity**  naturbete* AND biodiversitet  alvarmark* AND biodiversitet  alvarbete* AND biodiversitet  strandbete* AND biodiversitet  naturbete* AND "biologisk mångfald"  alvarmark* AND "biologisk mångfald"  alvarbete* AND "biologisk mångfald"  strandbete* AND "biologisk mångfald"  **PECO 2 - Outcome: Changed nutrient status of the soils**  naturbete* AND näring*  alvarmark* AND näring*  alvarbete* AND näring*  strandbete* AND näring*  **PO - Outcome: Behavioural measures**  naturbete* AND beteende*  alvarmark* AND beteende*  alvarbete* AND beteende*  strandbete* AND beteende* | **3**  **0**  **0**  **0**  **23**  **3**  **0**  **1**  **12**  **2**  **0**  **1**  **1**  **1**  **0**  **0** |

* = Represents any group of characters, including no character
" " = Searches for an exact phrase

**Database: ProQuest Natural Science Collection**

Database provider: ProQuest

Date of search: January 19, 2021; September 18, 2023

Including: AGRICOLA; Agricultural Science database; Aquatic Sciences and Fisheries Abstracts; Biological Science database; Biological Science index; Earth, atmosphere & Aquatic Science database; Environmental Science database; Environmental Science index; Meteorological & Geoastrophysical Abstracts

**Search string for PECO 1 - Outcome: Biodiversity**

| **No** | **Search string** | **Number of hits** (September 18, 2023) |
| --- | --- | --- |
|  | **Population: Semi-natural pastures** |  |
| **1** | ti,ab,su(((("semi-natural" OR seminatural OR natural OR "species rich" OR "nutrient poor" OR extensive OR extensively OR unimproved OR unfertilized OR alpine OR mountain OR forest* OR wood* OR rough OR calcareous OR siliceous OR montan* OR boreal OR temperate OR shore OR coast OR coastal OR wet OR wetted OR flood*) NEAR/5 (pasture OR pastureland OR pastoral OR grassland OR "grass land" OR graz* OR meadow OR rangeland OR "range land")) OR (alvar* OR heath* OR moorland* OR "moor land*" OR dehesa* OR montado* OR "pastoral landscape")) AND (graz* OR pasture OR pastureland OR enclos* OR fenc* OR paddock)) | **35 287** |
|  | **Exposure: Giving the grazers access to additional nutrient sources** |  |
| **2** | ti,ab,su(ley OR leys OR (("nutrient rich" OR fertilized OR improved OR intensive OR intensively ) NEAR/3 (pasture OR pastureland OR pastoral OR grassland OR "grass land")) OR ((former OR ex OR previous OR previously OR abandon*) NEAR/3 (field OR farmland OR "farm land" OR "arable land" OR "agricultural land" OR "cultivated land" OR cropland OR "crop land")) OR ((heterogen* OR additional OR multi* OR mix* OR divers OR diverse OR adjacent OR neighbor OR neighbouring OR vary OR varying OR varied OR variable OR various OR variation) NEAR/5 (graz* OR pasture OR pastureland OR grassland OR "grass land" OR enclos* OR fenc* OR paddock OR habitat OR biotop)) OR ("grazing regime" OR "pasture regime" OR "pastoral regime" OR "stocking regime" OR "grazing management" OR "pasture management" OR "pastoral management" OR "stocking management" OR "grazing strateg*" OR "pasture strateg*" OR "pastoral strateg*" OR "stocking strateg*" OR "grazing system" OR "pasture system" OR "pastoral system" OR "stocking system" OR "grazing method" OR "pasture method" OR "pastoral method" OR "stocking method" OR "grazing practice" OR "pasture practice" OR "pastoral practice" OR "stocking practice" OR "grazing experiment" OR "pasture experiment" OR "pastoral experiment" OR "stocking experiment" OR "pasture distribution" OR "field management" OR mosaic* OR "same enclosure" OR ranching OR "ranch farming") OR ((supply OR supplement OR supplemental OR enrich* OR additional OR creep* OR indoor* OR concentrate*) AND (feed* OR food OR nutrition OR nutritional OR fodder OR forage OR silage OR roughage OR hay OR mineral))) | **883 198** |
|  | **Outcome: Biodiversity** |  |
| **3** | ti,ab,su(biodiversity OR "bio-diversity" OR "biological diversity" OR ((species OR insect OR plant OR vegetation OR botanic OR botanical OR flora OR floral OR floristic OR fauna OR faunal OR faunistic OR invertebrete OR vertebrate OR organism OR functional OR taxa OR toxonomy OR toxonomic OR toxonomical OR trait) NEAR/5 (diversity OR abundant OR abundance OR richness OR heterogen* OR indicator OR frequen* OR composition OR distribution OR density OR dispers OR disperse OR dispersal OR target OR focal OR keystone OR umbrella OR "red-list" OR "red-listed" OR redlist OR redlisted OR threaten OR threatened OR endangered OR rare))) | **1 544 490** |
|  | **Combination of search strings** |  |
| **4** | 1 AND 2 AND 3 | **3 439** |
|  | **Limit to language: English, Danish, French, German, Norwegian, Spanish, Swedish** |  |
| **5** | AND la.exact("ENG" OR "DAN" OR "FRE" OR "GER" OR "NOR" OR "SPA" OR "SWE") | **3 039** |

**Search string for PECO 2 - Outcome: Changed nutrient status of the soils**

| **No** | **Search string** | **Number of hits** (September 18, 2023) |
| --- | --- | --- |
|  | **Population: Semi-natural pastures** |  |
| **1** | ti,ab,su(((("semi-natural" OR seminatural OR natural OR "species rich" OR "nutrient poor" OR extensive OR extensively OR unimproved OR unfertilized OR alpine OR mountain OR forest* OR wood* OR rough OR calcareous OR siliceous OR montan* OR boreal OR temperate OR shore OR coast OR coastal OR wet OR wetted OR flood*) NEAR/5 (pasture OR pastureland OR pastoral OR grassland OR "grass land" OR graz* OR meadow OR rangeland OR "range land")) OR (alvar* OR heath* OR moorland* OR "moor land*" OR dehesa* OR montado* OR "pastoral landscape")) AND (graz* OR pasture OR pastureland OR enclos* OR fenc* OR paddock)) | **35 287** |
|  | **Exposure: Giving the grazers access to additional nutrient sources** |  |
| **2** | ti,ab,su(ley OR leys OR (("nutrient rich" OR fertilized OR improved OR intensive OR intensively ) NEAR/3 (pasture OR pastureland OR pastoral OR grassland OR "grass land")) OR ((former OR ex OR previous OR previously OR abandon*) NEAR/3 (field OR farmland OR "farm land" OR "arable land" OR "agricultural land" OR "cultivated land" OR cropland OR "crop land")) OR ((heterogen* OR additional OR multi* OR mix* OR divers OR diverse OR adjacent OR neighbor OR neighbouring OR vary OR varying OR varied OR variable OR various OR variation) NEAR/5 (graz* OR pasture OR pastureland OR grassland OR "grass land" OR enclos* OR fenc* OR paddock OR habitat OR biotop)) OR ("grazing regime" OR "pasture regime" OR "pastoral regime" OR "stocking regime" OR "grazing management" OR "pasture management" OR "pastoral management" OR "stocking management" OR "grazing strateg*" OR "pasture strateg*" OR "pastoral strateg*" OR "stocking strateg*" OR "grazing system" OR "pasture system" OR "pastoral system" OR "stocking system" OR "grazing method" OR "pasture method" OR "pastoral method" OR "stocking method" OR "grazing practice" OR "pasture practice" OR "pastoral practice" OR "stocking practice" OR "grazing experiment" OR "pasture experiment" OR "pastoral experiment" OR "stocking experiment" OR "pasture distribution" OR "field management" OR mosaic* OR "same enclosure" OR ranching OR "ranch farming") OR ((supply OR supplement OR supplemental OR enrich* OR additional OR creep* OR indoor* OR concentrate*) AND (feed* OR food OR nutrition OR nutritional OR fodder OR forage OR silage OR roughage OR hay OR mineral))) | **883 211** |
|  | **Outcome: Changed nutrient status of the soils** |  |
| **3** | ti,ab,su((soil AND (nutrient* OR "nutritional status" OR "nutritional level" OR "nutritional value" OR phosphor* OR phosphate OR nitrogen* OR nitrate OR ammonium* OR potassium* OR calcium* OR magnesium*)) OR ("soil chemistry" OR "soil fertility" OR "soil condition*" OR "soil propert*" OR "plant indicator" OR ellenberg OR "biomass production" OR eutrophi* OR trophic* OR oligotrophi*)) | **733 099** |
|  | **Combination of search strings** |  |
| **4** | 1 AND 2 AND 3 | **1 504** |
|  | **Limit to language: English, Danish, French, German, Norwegian, Spanish, Swedish** |  |
| **5** | AND la.exact("ENG" OR "DAN" OR "FRE" OR "GER" OR "NOR" OR "SPA" OR "SWE") | **1 335** |

**Search string for PO - Outcome: Behavioural measures**

| **No** | **Search string** | **Number of hits** (September 18, 2023) |
| --- | --- | --- |
|  | **Population: Grazing domestic animals in semi-natural or natural pastures, that also have access to an additional nutrient source** |  |
| **1** | ti,ab,su((graz* OR herbivor* OR ruminant OR cattle OR livestock OR cow OR bull OR bulls OR beef OR sheep OR horse OR donkey OR mule OR goat OR lama OR llama OR alpaca) AND (((("semi-natural" OR seminatural OR natural OR "species rich" OR "nutrient poor" OR extensive OR extensively OR unimproved OR unfertilized OR alpine OR mountain OR forest* OR wood* OR rough OR calcareous OR siliceous OR montan* OR boreal OR temperate OR shore OR coast OR coastal OR wet OR wetted OR flood*) NEAR/5 (pasture OR pastureland OR pastoral OR grassland OR "grass land" OR graz* OR meadow OR rangeland OR "range land")) OR (alvar* OR heath* OR moorland* OR "moor land*" OR dehesa* OR montado* OR "pastoral landscape")) AND (graz* OR pasture OR pastureland OR enclos* OR fenc* OR paddock) AND (ley OR leys OR (("nutrient rich" OR fertilized OR improved OR intensive OR intensively ) NEAR/3 (pasture OR pastureland OR pastoral OR grassland OR "grass land")) OR ((former OR ex OR previous OR previously OR abandon*) NEAR/3 (field OR farmland OR "farm land" OR "arable land" OR "agricultural land" OR "cultivated land" OR cropland OR "crop land")) OR ((heterogen* OR additional OR multi* OR mix* OR divers OR diverse OR adjacent OR neighbor OR neighbouring OR vary OR varying OR varied OR variable OR various OR variation) NEAR/5 (graz* OR pasture OR pastureland OR grassland OR "grass land" OR enclos* OR fenc* OR paddock OR habitat OR biotop)) OR ("grazing regime" OR "pasture regime" OR "pastoral regime" OR "stocking regime" OR "grazing management" OR "pasture management" OR "pastoral management" OR "stocking management" OR "grazing strateg*" OR "pasture strateg*" OR "pastoral strateg*" OR "stocking strateg*" OR "grazing system" OR "pasture system" OR "pastoral system" OR "stocking system" OR "grazing method" OR "pasture method" OR "pastoral method" OR "stocking method" OR "grazing practice" OR "pasture practice" OR "pastoral practice" OR "stocking practice" OR "grazing experiment" OR "pasture experiment" OR "pastoral experiment" OR "stocking experiment" OR "pasture distribution" OR "field management" OR mosaic* OR "same enclosure" OR ranching OR "ranch farming") OR ((supply OR supplement OR supplemental OR enrich* OR additional OR creep* OR indoor* OR concentrate*) AND (feed* OR food OR nutrition OR nutritional OR fodder OR forage OR silage OR roughage OR hay OR mineral))))) | **7 613** |
|  | **Outcome: Behavioural measures** |  |
| **2** | ti,ab,su(behaviour OR behavioural OR behavior OR behavioral OR move OR movement OR moving OR trample OR trampling OR tread OR treading OR scrape OR scraping OR urin OR urinate OR urinating OR excret* OR defecat* OR feces OR faeces OR dung OR dunging OR manure OR fouling OR "stock distribution" OR "stocking distribution" OR "livestock distribution" OR "herd distribution" OR "grazing distribution" OR "grazing pressure" OR "grazing pattern" OR "grazing time" OR "grazing period" OR "grazing intensity" OR "grazing habit" OR "grazing habits" OR "under-graz*" OR undergraz* OR "over-graz*" OR overgraz* OR foraging OR "resting time" OR "resting period") OR ((graz* OR diet OR eat OR eating OR feed OR feeding OR food OR fodder OR forage OR foraging) NEAR/5 (select OR selection OR prefer OR preference OR choose OR choice)) | **6 121 116** |
|  | **Combination of search strings** |  |
| **3** | 1 AND 2 | **2 116** |
|  | **Limit to language: English, Danish, French, German, Norwegian, Spanish, Swedish** |  |
| **4** | AND la.exact("ENG" OR "DAN" OR "FRE" OR "GER" OR "NOR" OR "SPA" OR "SWE") | **1 888** |

* = Represents any group of characters, including no character (Formas ProQuest settings is configured to automatically search for the plural forms of the search terms)
" " = Searches for an exact phrase
ti,ab,su = Title or Abstract or All subjects & indexing
la.exact = Language

NEAR/5 = A proximity operator to find terms within five words from each other

NEAR/3 = A proximity operator to find terms within three words from each other

**Database: SwePub**
Database provider: National Library of Sweden
Date of search: January 20, 2021; September 19, 2023

SwePub contains references to articles, conference papers and dissertations published at Swedish universities and authorities.

| **Language** | **Search string** | **Number of hits** (September 19, 2023) |
| --- | --- | --- |
| **Search string for PECO 1 - Outcome: Biodiversity** | | |
| **English** | ("semi-natural" OR seminatural) AND (pasture* OR grassland* OR "grass land*") AND (biodiversity OR "bio-diversity" OR "biological diversity") | **168** |
| **Swedish** | ("semi-natur*" OR seminatur* OR naturbete* OR alvarmark* OR alvarbete* OR strandbete*) AND (biodiversitet OR "biologisk mångfald") | **101** |
| **Search string for PECO 2 - Outcome: Changed nutrient status of the soils** | | |
| **English** | ("semi-natural" OR seminatural) AND (pasture* OR grassland* OR "grass land*") AND nutri* AND soil* | **16** |
| **Swedish** | ("semi-natur*" OR seminatur* OR naturbete* OR alvarmark* OR alvarbete* OR strandbete*) AND näring* | **41** |
| **Search string for PO - Outcome: Behavioural measures** | | |
| **English** | ("semi-natural" OR seminatural) AND (pasture* OR grassland* OR "grass land*") AND (behav* OR mov*) | **21** |
| **Swedish** | ("semi-natur*" OR seminatur* OR naturbete* OR alvarmark* OR alvarbete* OR strandbete*) AND beteende* | **4** |

* = Represents any group of characters, including no character
" " = Searches for an exact phrase

**Search engine**

**Google Scholar**

Date of search: January 20, 2021; September 18, 2023
The first 50 results for every search will be exported from Google Scholar using Publish or Perish version 6 software: Harzing, A.W. (2007) Publish or Perish, available from https://harzing.com/resources/publish-or-perish

| **Language** | **Search string** | **Number of hits** (September 18, 2023) |
| --- | --- | --- |
| **English** | **Biodiversity**  Any of the words: pasture grassland  All of the words: "semi-natural" biodiversity  **Changed nutrient status** Any of the words: pasture grassland  All of the words: "semi-natural" nutrient  **Behavioural measures**  Any of the words: behaviour behavior  All of the words: "semi-natural" pasture  Any of the words: behaviour behavior  All of the words: "semi-natural" grassland | **50**  **50**  **50**  **50** |
| **Danish** | **Biodiversity**  Any of the words: overdrev eng hede strandeng  All of the words: biodiversitet  Any of the words: overdrev eng hede strandeng  All of the words: "biologisk mangfoldighed"  **Changed nutrient status** Any of the words: overdrev eng hede strandeng  All of the words: næring  Any of the words: overdrev eng hede strandeng  All of the words: næringsstof  Any of the words: overdrev eng hede strandeng  All of the words: kvælstof  **Behavioural measures**  Any of the words: overdrev eng hede strandeng  All of the words: adfærd | **50**  **50**  **50**  **50**  **50**  **50** |
| **Norwegian** | **Biodiversity**  Any of the words: naturbeitemark "semi-naturlig eng" "semi-naturlig strandeng" hagemark kulturmarkseng  All of the words: "biologisk mangfold"  Any of the words: naturbeitemark "semi-naturlig eng" "semi-naturlig strandeng" hagemark kulturmarkseng  All of the words: biomangfold  Any of the words: naturbeitemark "semi-naturlig eng" "semi-naturlig strandeng" hagemark kulturmarkseng  All of the words: biodiversitet  **Changed nutrient status** Any of the words: naturbeitemark "semi-naturlig eng" "semi-naturlig strandeng" hagemark kulturmarkseng  All of the words: næring  Any of the words: naturbeitemark "semi-naturlig eng" "semi-naturlig strandeng" hagemark kulturmarkseng  All of the words: nitrogen  **Behavioural measures**  Any of the words: naturbeitemark "semi-naturlig eng" "semi-naturlig strandeng" hagemark kulturmarkseng  All of the words: atferd  Any of the words: naturbeitemark "semi-naturlig eng" "semi-naturlig strandeng" hagemark kulturmarkseng  All of the words: adferd | **50**  **50**  **50**  **50**  **50**  **39**  **50** |
| **Swedish** | **Biodiversity**  Any of the words: naturbetesmarker naturbete alvarmark strandbete  All of the words: "biologisk mångfald"  Any of the words: naturbetesmarker naturbete alvarmark strandbete  All of the words: biodiversitet  **Changed nutrient status** Any of the words: naturbetesmarker naturbete alvarmark strandbete  All of the words: näring  Any of the words: naturbetesmarker naturbete alvarmark strandbete  All of the words: näringsstatus  **Behavioural measures**  Any of the words: naturbetesmarker naturbete alvarmark strandbete  All of the words: beteende | **50**  **50**  **50**  **50**  **50** |

" " = Searches for an exact phrase
No truncation (*) is used because Google Scholar automatically searches plural forms of the search terms

**Websites of relevant organizations**

| **Website** | **Date** | **Search string** | **Number of potentially relevant documents** |
| --- | --- | --- | --- |
| bioRxiv (online archive for unpublished preprints in biology) https://www.biorxiv.org | June 4, 2021 | **Used Advanced Search Search field: Abstract or Title Search mode: All words**  "semi-natural" pasture*  "semi-natural" grassland*  "semi-natural" "grass land*" | **2**  **10**  **0** |
| Conservation Evidence  http://www.conservationevidence.com | June 4, 2021 | **Used search field for Studies**  "semi-natural" pasture*  "semi-natural" grassland*  "semi-natural" "grass land*" | **47**  **29**  **0** |
| European chapter of the Society for Ecological Restoration (SER)  http://chapter.ser.org/europe | February 2, 2022 | Could not search due to database error in their Knowledge Base  https://chapter.ser.org/europe/knowledge-base/ | **0** |
| European Commission Joint Research Centre  http://ec.europa.eu/jrc | June 17, 2021 | **Used the search box on the publications page**  **https://ec.europa.eu/jrc/en/publications-list**  "semi-natural" AND pasture*  "semi-natural" AND grassland*  "semi-natural" AND "grass land*"  graz* AND pasture*  graz* AND grassland*  dupligraz* AND "grass land*" | **1**  **3**  **0**  **1**  **6**  **0** |
| European Environment Agency  http://www.eea.europa.eu | July 9, 2021 | **Used the search box on the publications page https://www.eea.europa.eu/publications**  "semi-natural"  graz* | **3**  **0** |
| Danmarks Miljøportal (Environmental Portal of Denmark)  https://miljoeportal.dk | August 30, 2023 | **Used the search box on the page: https://miljoeportal.dk**  Omdrev Biodiversitet  Eng Biodiversitet  Hede Biodiversitet | **0** **3** **0** |
| Landbrugsstyrelsen (Danish Agricultural Agency)  https://lbst.dk | August 30, 2023 | **Used the search box on the page: https://lbst.dk**  Omdrev Biodiversitet  Eng Biodiversitet  Hede Biodiversitet | **20** (only first 20 hits scanned) **20** (only first 20 hits scanned) **20** (only first 20 hits scanned) |
| Miljøstyrelsen (Danish  Environmental Protection Agency)  https://mst.dk | August 30, 2023 | **Used the search box on the page: https://mst.dk**  Omdrev Biodiversitet  Eng Biodiversitet  Hede Biodiversitet | **20** (only first 20 hits scanned) **20** (only first 20 hits scanned) **20** (only first 20 hits scanned) |
| Ministry of the Environment in Denmark  https://mim.dk | August 30, 2023 | **Used the search box on the page: https://mim.dk**  Omdrev Biodiversitet  Eng Biodiversitet  Hede Biodiversitet | **20** (only first 20 hits scanned) **20** (only first 20 hits scanned) **20** (only first 20 hits scanned) |
| Ministry of Food, Agriculture and Fisheries of Denmark  https://fvm.dk | August 30, 2023 | **Used the search box on the page: https://fvm.dk**  Omdrev Biodiversitet Eng Biodiversitet  Hede Biodiversitet | **20** (only first 20 hits scanned) **20** (only first 20 hits scanned) **20** (only first 20 hits scanned) |
| Luke (Natural Resources In0stitute of Finland)  https://www.luke.fi | February 2, 2022 | **Used Advanced search in their Jukuri publication service https://jukuri.luke.fi/discover?query=&scope=**  "semi-natural" AND pasture  "semi-natural" AND grassland  "semi-natural" AND "grass land"  semi-naturlig  naturbete* | **4**  **6**  **1**  **0**  **0** |
| Metsähallitus (Steward of state-owned land and water areas in Finland)  https://www.metsa.fi | February 8, 2022 | **Used the free text search box on their publications site**  **https://julkaisut.metsa.fi**  semi-natural  pasture  pastures  grassland  grasslands  grass land  grass lands | **2**  **0**  **0**  **0**  **0**  **0**  **0** |
| Ministry of Agriculture and Forestry in Finland  https://mmm.fi | February 8, 2022 | **Used the search box on the publications page**  **https://mmm.fi/sv/publikationer**  semi-natural  pasture  pastures  grassland  grasslands  grass land  grass lands  semi-naturlig  naturbete  naturbetesmark  betesmark | **0**  **0**  **0**  **0**  **0**  **0**  **0**  **0**  **0**  **0**  **0** |
| Ministry of the Environment in Finland  https://ym.fi | February 8, 2022 | **Used the search box on the publications page**  **https://ym.fi/sv/publikationer**  semi-natural  pasture  pastures  grassland  grasslands  grass land  grass lands  semi-naturlig  naturbete  naturbetesmark  betesmark | **0**  **2**  **0**  **0**  **0**  **0**  **0**  **0**  **0**  **0**  **0** |
| SYKE (Finnish Environment Institute)  https://www.syke.fi | February 14, 2022 | **Used simple search in their publications archive**  **https://helda.helsinki.fi/handle/10138/29865?locale-attribute=en**  "semi-natural" AND pasture  "semi-natural" AND grassland  "semi-natural" AND "grass land"  "semi-naturlig"  naturbete* | **7**  **1**  **2**  **0**  **2** |
| BioFokus (Norway)  https://biofokus.no | July 27, 2023 | **Used simple search in their publications archive** [**https://biofokus.no/publikasjoner/**](https://biofokus.no/publikasjoner/)  naturbeitemark  beitemark  hagemark  semi-naturlig eng  semi-naturlig strandeng  kulturmarkseng  kulturmark | **4**  **7**  **0**  **1**  **0**  **0**  **4** |
| Landbruksdirektoratet (Norwegian Agricultural Agency)  https://www.landbruksdirektoratet.no | July 27, 2023 | **Used simple search in their publications archive** [**https://www.landbruksdirektoratet.no/nb/nyhetsrom/rapporter**](https://www.landbruksdirektoratet.no/nb/nyhetsrom/rapporter)  naturbeitemark  beitemark  hagemark  semi-naturlig eng  semi-naturlig strandeng  kulturmarkseng  kulturmark | **0**  **0**  **0**  **4**  **0**  **0**  **0** |
| Miljødirektoratet (Norwegian Environment Agency)  https://www.miljodirektoratet.no | July 27, 2023 | **Used simple search in their publications archive** [**https://www.miljodirektoratet.no/publikasjoner/**](https://www.miljodirektoratet.no/publikasjoner/)  naturbeitemark  beitemark  hagemark  semi-naturlig eng  semi-naturlig strandeng  kulturmarkseng  kulturmark | **1**  **0**  **0**  **19**  **9**  **0**  **1** |
| Ministry of Agriculture and Food in Norway  https://www.regjeringen.no/no/dep/lmd/id627 | July 27, 2023 | **Used simple search in their publications archive** [**https://www.regjeringen.no/no/dokument/rapportar-og-planar/id438817/?ownerid=627&term**](https://www.regjeringen.no/no/dokument/rapportar-og-planar/id438817/?ownerid=627&term) **(filtered by ministry)**  naturbeitemark  beitemark  hagemark  semi-naturlig eng  semi-naturlig strandeng  kulturmarkseng  kulturmark | **0**  **0**  **0**  **1**  **0**  **0**  **0** |
| NIBIO (Norwegian Institute of Bioeconomy Research)  https://www.nibio.no | July 27, 2023 | **Used simple search in their publications archive** [**https://www.nibio.no/publikasjoner**](https://www.nibio.no/publikasjoner)  naturbeitemark  beitemark  hagemark  “semi-naturlig eng”  “semi-naturlig strandeng”  kulturmarkseng  kulturmark | **62**  **48**  **22**  **12**  **1**  **11**  **104** |
| NINA (Norwegian Institute for Nature Research)  https://www.nina.no | July 27, 2023 | **Used simple search in their publications archive** [**https://www.nibio.no/publikasjoner**](https://www.nibio.no/publikasjoner)  naturbeitemark  beitemark  hagemark  “semi-naturlig eng”  “semi-naturlig strandeng”  kulturmarkseng  kulturmark | **12**  **31**  **12**  **20**  **4**  **6**  **17** |
| Jordbruksverket (Swedish Board of Agriculture)  https://jordbruksverket.se | February 15, 2022 | **Used the search box on the publications page https://webbutiken.jordbruksverket.se/**  semi-naturlig  naturbete  naturbetesmark  betesmark | **0**  **9**  **0**  **21** |
| Länsstyrelsen Blekinge (County Administrative Board of Blekinge, Sweden) https://www.lansstyrelsen.se/blekinge | February 18, 2022 | **Used the search box on the publications page**  **https://www.lansstyrelsen.se/blekinge/om-oss/vara-tjanster/publikationer.html**  semi-naturlig*  naturbete*  betesmark*  biologisk mångfald bete* | **0**  **0**  **1**  **0** |
| Länsstyrelsen Dalarna (County Administrative Board of Dalarna, Sweden) https://www.lansstyrelsen.se/dalarna | February 18, 2022 | **Used the search box on the publications page**  **https://www.lansstyrelsen.se/dalarna/om-oss/vara-tjanster/publikationer.html**  semi-naturlig*  naturbete*  betesmark*  biologisk mångfald bete* | **0**  **0**  **0**  **0** |
| Länsstyrelsen Gotland (County Administrative Board of Gotland, Sweden) https://www.lansstyrelsen.se/gotland | February 18, 2022 | **Used the search box on the publications page**  **https://www.lansstyrelsen.se/gotland/om-oss/vara-tjanster/publikationer.html**  semi-naturlig*  naturbete*  betesmark*  biologisk mångfald bete* | **0**  **0**  **3**  **0** |
| Länsstyrelsen Gävleborg (County Administrative Board of Gävleborg, Sweden) https://www.lansstyrelsen.se/gavleborg | February 23, 2022 | **Used the search box on the publications page**  **https://www.lansstyrelsen.se/gavleborg/om-oss/vara-tjanster/publikationer.html**  semi-naturlig*  naturbete*  betesmark*  biologisk mångfald bete* | **0**  **0**  **0**  **0** |
| Länsstyrelsen Halland (County Administrative Board of Halland, Sweden) https://www.lansstyrelsen.se/halland | February 23, 2022 | **Used the search box on the publications page**  **https://www.lansstyrelsen.se/halland/om-oss/vara-tjanster/publikationer.html**  semi-naturlig*  naturbete*  betesmark*  biologisk mångfald bete* | **0**  **1**  **1**  **0** |
| Länsstyrelsen Jämtland (County Administrative Board of Jämtland, Sweden) https://www.lansstyrelsen.se/jamtland | February 23, 2022 | **Used the search box on the publications page**  **https://www.lansstyrelsen.se/jamtland/om-oss/vara-tjanster/publikationer.html**  semi-naturlig*  naturbete*  betesmark*  biologisk mångfald bete* | **0**  **0**  **1**  **0** |
| Länsstyrelsen Jönköping (County Administrative Board of Jönköping, Sweden) https://www.lansstyrelsen.se/jonkoping | February 23, 2022 | **Used the search box on the publications page**  **https://www.lansstyrelsen.se/jonkoping/om-oss/vara-tjanster/publikationer.html**  semi-naturlig*  naturbete*  betesmark*  biologisk mångfald bete* | **0**  **0**  **2**  **0** |
| Länsstyrelsen Kalmar (County Administrative Board of Kalmar, Sweden) https://www.lansstyrelsen.se/kalmar | February 23, 2022 | **Used the search box on the publications page**  **https://www.lansstyrelsen.se/kalmar/om-oss/vara-tjanster/publikationer.html**  semi-naturlig*  naturbete*  betesmark*  biologisk mångfald bete* | **0**  **0**  **1**  **1** |
| Länsstyrelsen Kronoberg (County Administrative Board of Kronoberg, Sweden) https://www.lansstyrelsen.se/kronoberg | February 23, 2022 | **Used the search box on the publications page**  **https://www.lansstyrelsen.se/kronoberg/om-oss/vara-tjanster/publikationer.html**  semi-naturlig*  naturbete*  betesmark*  biologisk mångfald bete* | **0**  **0**  **0**  **0** |
| Länsstyrelsen Norrbotten (County Administrative Board of Norrbotten, Sweden) https://www.lansstyrelsen.se/norrbotten | February 23, 2022 | **Used the search box on the publications page**  **https://www.lansstyrelsen.se/norrbotten/om-oss/vara-tjanster/publikationer.html**  semi-naturlig*  naturbete*  betesmark*  biologisk mångfald bete* | **0**  **0**  **1**  **1** |
| Länsstyrelsen Skåne (County Administrative Board of Skåne, Sweden) https://www.lansstyrelsen.se/skane | February 23, 2022 | **Used the search box on the publications page**  **https://www.lansstyrelsen.se/skane/om-oss/vara-tjanster/publikationer.html**  semi-naturlig*  naturbete*  betesmark*  biologisk mångfald bete* | **0**  **1**  **5**  **1** |
| Länsstyrelsen Stockholm (County Administrative Board of Stockholm, Sweden) https://www.lansstyrelsen.se/stockholm | February 24, 2022 | **Used the search box on the publications page**  **https://www.lansstyrelsen.se/stockholm/om-oss/vara-tjanster/publikationer.html**  semi-naturlig*  naturbete*  betesmark*  biologisk mångfald bete* | **0**  **0**  **1**  **0** |
| Länsstyrelsen Södermanland (County Administrative Board of Södermanland, Sweden) <https://www.lansstyrelsen.se/sodermanland> | February 24, 2022 | **Used the search box on the publications page**  **https://www.lansstyrelsen.se/sodermanland/om-oss/vara-tjanster/publikationer.html**  semi-naturlig*  naturbete*  betesmark*  biologisk mångfald bete* | **0**  **0**  **1**  **0** |
| Länsstyrelsen Uppsala (County Administrative Board of Uppsala, Sweden) https://www.lansstyrelsen.se/uppsala | February 24, 2022 | **Used the search box on the publications page**  **https://www.lansstyrelsen.se/uppsala/om-oss/vara-tjanster/publikationer.html**  semi-naturlig*  naturbete*  betesmark*  biologisk mångfald bete* | **0**  **0**  **0**  **0** |
| Länsstyrelsen Värmland (County Administrative Board of Värmland, Sweden) https://www.lansstyrelsen.se/varmland | February 24, 2022 | **Used the search box on the publications page**  **https://www.lansstyrelsen.se/varmland/om-oss/vara-tjanster/publikationer.html**  semi-naturlig*  naturbete*  betesmark*  biologisk mångfald bete* | **0**  **0**  **1**  **0** |
| Länsstyrelsen Västerbotten (County Administrative Board of Västerbotten, Sweden) https://www.lansstyrelsen.se/vasterbotten | February 24, 2022 | **Used the search box on the publications page**  **https://www.lansstyrelsen.se/vasterbotten/om-oss/vara-tjanster/publikationer.html**  semi-naturlig*  naturbete*  betesmark*  biologisk mångfald bete* | **0**  **0**  **0**  **0** |
| Länsstyrelsen Västernorrland (County Administrative Board of Västernorrland, Sweden) https://www.lansstyrelsen.se/vasternorrland | February 24, 2022 | **Used the search box on the publications page**  **https://www.lansstyrelsen.se/vasternorrland/om-oss/vara-tjanster/publikationer.html**  semi-naturlig*  naturbete*  betesmark*  biologisk mångfald bete* | **0**  **0**  **0**  **0** |
| Länsstyrelsen Västmanland (County Administrative Board of Västmanland, Sweden) https://www.lansstyrelsen.se/vastmanland | February 24, 2022 | **Used the search box on the publications page**  **https://www.lansstyrelsen.se/vastmanland/om-oss/vara-tjanster/publikationer.html**  semi-naturlig*  naturbete*  betesmark*  biologisk mångfald bete* | **0**  **0**  **1**  **0** |
| Länsstyrelsen Västra Götaland (County Administrative Board of Västra Götaland, Sweden) https://www.lansstyrelsen.se/vastra-gotaland | February 25, 2022 | **Used the search box on the publications page**  **https://www.lansstyrelsen.se/vastra-gotaland/om-oss/vara-tjanster/publikationer.html**  semi-naturlig*  naturbete*  betesmark*  biologisk mångfald bete* | **0**  **1**  **9**  **0** |
| Länsstyrelsen Örebro (County Administrative Board of Örebro, Sweden) https://www.lansstyrelsen.se/orebro | February 25, 2022 | **Used the search box on the publications page**  **https://www.lansstyrelsen.se/orebro/om-oss/vara-tjanster/publikationer.html**  semi-naturlig*  naturbete*  betesmark*  biologisk mångfald bete* | **0**  **2**  **2**  **0** |
| Länsstyrelsen Östergötland (County Administrative Board of Östergötland, Sweden) https://www.lansstyrelsen.se/ostergotland | February 25, 2022 | **Used the search box on the publications page**  **https://www.lansstyrelsen.se/ostergotland/om-oss/vara-tjanster/publikationer.html**  semi-naturlig*  naturbete*  betesmark*  biologisk mångfald bete* | **0**  **0**  **4**  **0** |
| Naturvårdsverket (Swedish Environmental Protection Agency) <http://www.naturvardsverket.se> | February 16, 2022 | **Used Simple search in their publication service https://naturvardsverket.diva-portal.org/**  semi-naturlig*  naturbete*  betesmark*  "biologisk mångfald" AND bete* | **0**  **15**  **9**  **4** |
| SLU (Swedish University of Agricultural Sciences) <https://www.slu.se> | February 25, 2022 | **Used Advanced search in their publication service Epsilon https://pub.epsilon.slu.se/cgi/search/advanced Search field: Abstract, all of the words**  "semi-natural" pasture*  "semi-natural" grassland*  "semi-natural" "grass land*"  graz* pasture*  graz* grassland*  graz* "grass land*"  "semi-naturlig*"  naturbete*  betesmark*  "biologisk mångfald" bete* | **16**  **11**  **0**  **4**  **5**  **3**  **3**  **14**  **12**  **1** |
| DEFRA (Department for Environment, Food & Rural Affairs)  http://randd.defra.gov.uk | March 7, 2022 | **Used the search box for completed and ongoing projects**  **http://randd.defra.gov.uk/Default.aspx?Location=None&Module=FilterSearchNewLook&Completed=0**  **Project Status: All**  "semi-natural"  grazing AND pasture  grazing AND grassland  grazing AND "grass land" | **2**  **0**  **3**  **0** |
| Natural England  http://publications.naturalengland.org.uk | March 7, 2022 | **Browsed through the publication lists on the site** | **7** |
| Natural Resources Wales  http://libcat.naturalresources.wales | March 8, 2022 | **Used the Keyword search**  "semi-natural" AND pasture  "semi-natural" AND grassland  grazing AND pasture  grazing AND grassland | **0**  **5**  **2**  **2** |
| NatureScot (Scotland's Nature Agency)  https://www.nature.scot | March 8, 2022 | **Used the regular search box for the entire website**  "semi-natural" AND pasture  "semi-natural" AND grassland  grazing AND pasture  grazing AND grassland | **1**  **2**  **4**  **2** |
| NORA (Research publications from British Antarctic Survey, British Geological Survey, National Oceanography Centre, and UK Centre for Ecology & Hydrology)  http://nora.nerc.ac.uk | March 9, 2022 | **Used Advanced search Search field: Title, all of the words**  "semi-natural"  grazing pasture  grazing grassland  grazing "grass land" | **19**  **0**  **10**  **0** |
| UK Environment Agency  https://www.gov.uk/government/publications?departments%5B%5D=environment-agency | March 9, 2022 | **Used the regular search box for the entire website**  **Limit to Content type: "Research and statistics"**  "semi-natural"  grazing AND pasture  grazing AND grassland | **0**  **0**  **0** |
