# Supplementary material for: Effects on biodiversity in semi-natural pastures of giving the grazing animals access to additional nutrient sources: a systematic review
Source: Environ Evid. 2024 Aug 1;13:18. doi: 10.1186/s13750-024-00343-4 (PMC11378873; doi:10.1186/s13750-024-00343-4)
Supplement: Supplementary file 3 — Additional file 3: Reviews checked for relevant references. [file 13750_2024_343_MOESM3_ESM.docx]

README

Title: Reviews checked for references

Description: This additional file contains a list of relevant review articles that we have gone through to find potentially relevant primary studies.

# Reviews that have been checked for potentially relevant references

Bailey, D. W., Mosley, J. C., Estell, R. E., Cibils, A. F., Horney, M., Hendrickson, J. R., ... & Burritt, E. A. (2019). Synthesis paper: targeted livestock grazing: prescription for healthy rangelands. *Rangeland Ecology & Management*, *72*(6), 865-877.

Badgery, W. B., & Michalk, D. L. (2017). Synthesis of system outcomes for a grazing-management experiment in temperate native pastures. *Animal production science*, 57(9), 1869-1876.

Bohnert, D. W., & Stephenson, M. B. (2016). Supplementation and sustainable grazing systems. *Journal of Animal Science*, 94, 15-25.

Celaya, R., Ferreira, L. M. M., Lorenzo, J. M., Echegaray, N., Crecente, S., Serrano, E., & Busqué, J. (2022). Livestock Management for the Delivery of Ecosystem Services in Fire-Prone Shrublands of Atlantic Iberia. *Sustainability (Switzerland),* 14(5).

Creamer, M. L., Roche, L. M., Horback, K. M., & Saitone, T. L. (2019). Optimising cattle grazing distribution on rangeland: a systematic review and network analysis. *Rangeland Journal*, 41(5), 441-455.

Critchley, C. N. R., Burke, M. J. W., & Stevens, D. P. (2004). Conservation of lowland semi-natural grasslands in the UK: a review of botanical monitoring results from agri-environment schemes. *Biological Conservation*, 115(2), 263-278.

Dahlström, F., Hessle, A., & Kumm, K. I. (2018). Bete i skog som en foderresurs. pub.epsilon.slu.se. https://pub.epsilon.slu.se/15599/11/dahlstrom_f_et_al_180918.pdf

Durant, D., Tichit, M., Kerneis, E., & Fritz, H. (2008). Management of agricultural wet grasslands for breeding waders: integrating ecological and livestock system perspectives - a review. *Biodiversity and Conservation*, 17(9), 2275-2295.

Gomez-Garcia, D., Garcia-Gonzalez, R., & Fillat, F. Multifunctionality of mountain grasslands: towards a multidisciplinar interpretation of grassland systems in the Pyrenees of Aragon. In (pp. XLVIII Reunion cientifica de la Sociedad Espanola para el Estudio de los Pastos, Huesca, Spain, 15-18 junio 2009; 2009 2015-2041 many ref). *La multifuncionalidad de los pastos Sociedad Espanola para el Estudio de los Pastos* (SEEP).

Hendricksen, R. E., McLean, R. W., & Dicker, R. W. (1985). The role of supplements and diet selection in beef production and management of tropical pastures in Australia. Proceedings of the third Australian Conference on Tropical Pastures, Rockhampton, Australia.

Kilgour, R. (1974). Potential value of animal behaviour studies in animal production. Proceedings of the Australian Society of Animal Production, 10, 286-298.

Kilgour, R. J. (2012). In pursuit of “normal”: A review of the behaviour of cattle at pasture. *Applied Animal Behaviour Science.* https://www.sciencedirect.com/science/article/pii/S0168159111004229

Laca, E. A. (2009). New approaches and tools for grazing management. (Special Feature: Understanding diet selection in temperate biodiverse pasture systems.). *Rangeland Ecology & Management*, 62(5), 407-417.

Masson, C., Rubino. R., & Fedele, V. (1991). Forage utilization in goats. *Goat nutrition*, 145-159. 31 ref.

Michalk, D. L., Badgery, W. B., & Kemp, D. R. (2017). Balancing animal, pasture and environmental outcomes in grazing management experiments. *Animal production science*, 57(9), 1775-1784.

Olff, H., Vera, F. W. M., Bokdam, J., Bakker, E. S., Gleichman, J. M., Maeyer, K. d., & Smit, R. (1999). Shifting mosaics in grazed woodlands driven by the alternation of plant facilitation and competition. *Plant Biology*, 1(2), 127-137.

Osoro, K., Ferreira, L. M. M., García, U., Rosa García, R., Martínez, A., & Celaya, R. (2012). Grazing systems and the role of horses in heathland areas. *EAAP Scientific Series*, 132(1), 137-146. https://doi.org/10.3920/978-90-8686-755-4_15

Peyraud, J. L., Delagarde, R., & Delaby, L. (1995). Effect of grazing management and animal characteristics on grazing intake by dairy cows: analysis and forecasting. 2nd Congress on Ruminant Research, Paris, France.

Scottish Natural Heritage (2016) ‘The effects of supplementary feeding on species-rich grassland’. SNH guidance note.

Soder, K. J., Gregorini, P., Scaglia, G., & Rook, A. J. (2009). Dietary selection by domestic grazing ruminants in temperate pastures: current state of knowledge, methodologies, and future direction. (Special Feature: Understanding diet selection in temperate biodiverse pasture systems.). *Rangeland Ecology & Management*, 62(5), 389-398.

Soder, K. J., Rook, A. J., Sanderson, M. A., & Goslee, S. C. (2007). Interaction of plant species diversity on grazing behavior and performance of livestock grazing temperate region pastures. *Crop Science*, 47(1), 416-425.

Tallowin, J. R. B. (1997). The agricultural productivity of lowland semi-natural grassland: a review.

Valko, O., Zmihorski, M., Biurrun, I., Loos, J., Labadessa, R., & Venn, S. (2016). Ecology and conservation of steppes and semi-natural grasslands. *Hacquetia*, 15(2), 5-14.

WallisDeVries, M. F. (2016). Grazing and biodiversity: from selective foraging to wildlife habitats. Options Mediterraneennes. Serie A, Seminaires Mediterraneens, 116, 177-187.

Whitehead, D. C. (2000). Nutrient elements in grassland: soil-plant-animal relationships / David C. Whitehead. CABI Pub.

Wilkinson, J. M., Lee, M. R. F., Rivero, M. J., & Chamberlain, A. T. (2020). Some challenges and opportunities for grazing dairy cows on temperate pastures. Grass and Forage Science, 75(1), 1-17.
