# Supplementary material for: Effects on biodiversity in semi-natural pastures of giving the grazing animals access to additional nutrient sources: a systematic review
Source: Environ Evid. 2024 Aug 1;13:18. doi: 10.1186/s13750-024-00343-4 (PMC11378873; doi:10.1186/s13750-024-00343-4)
Supplement: Supplementary file 4 — Additional file 4: Benchmark studies. [file 13750_2024_343_MOESM4_ESM.docx]

*README*

Title: Benchmark studies

Description: This additional file is a list of published studies that is relevant to the subject matter of the systematic review. These studies are called "benchmark studies" and this list was used during the development of the search strings and to test the comprehensiveness of the search.

**Benchmark studies**

It should be noted that it is not ensured that all of the listed benchmark studies fulfill the criteria to be included in the systematic review. However, they are all sufficiently relevant to be used to test the comprehensiveness of the search.

**Benchmark studies for PECO 1 and PECO 2**

Eriksson Å, Eriksson O, Berglund H. Species abundance patterns of plants in Swedish semi-natural pastures. Ecography 1995;18(3):310-317.

Pykälä J. Maintaining biodiversity through traditional animal husbandry. The Finnish Environment 2001;495:28-62.
*Not indexed in Scopus and therefore not found during the comprehensiveness test of the search.*

Takala T, Haverinen J, Kuusela E, Tahvanainen T, Kouki J. Does cattle movement between forest pastures and fertilized grasslands affect the bryophyte and vascular plant communities in vulnerable forest pasture biotopes? Agriculture, Ecosystems and Environment 2015;201:26-42.

van Uytvanck J, Milotic T, Hoffman M. [Nitrogen Depletion and Redistribution by Free‐Ranging Cattle in the Restoration Process of Mosaic Landscapes: The Role of Foraging Strategy and Habitat Proportion. Restoration Ecology](https://onlinelibrary.wiley.com/doi/full/10.1111/j.1526-100X.2009.00599.x?casa_token=sfPKzDh3a9IAAAAA%3AUiJajpe0Ze38tieIYx0U-I2WIOHqnf6iHTvoAkhBu9PWhuOiqjKFEIRn3Loon7xGzVi7vzUH8Oqf) 2010;18(1):205-216.

### **Benchmark studies for PO**

Hessle A, Rutter M, Wallin K. Effect of breed, season and pasture moisture gradient on foraging behavior in cattle on semi-natural grasslands. Applied Animal Behavior Science 2008;111:108-119.

Malkamäki E, Hæggström C-A. Short term impact of Finnish landrace cattle on the vegetation and soil of a wood pasture in SW Finland. Acta Botanica Fennica 1997;159:1-25.

Pelve ME, Spörndly E, Olsson I, Glimskär A. Grazing and fouling behaviour of cattle on different vegetation types within heterogeneous semi-natural and naturalised pastures. Livestock Science 2020;241:104253.
