# Supplementary material for: Effects on biodiversity in semi-natural pastures of giving the grazing animals access to additional nutrient sources: a systematic review
Source: Environ Evid. 2024 Aug 1;13:18. doi: 10.1186/s13750-024-00343-4 (PMC11378873; doi:10.1186/s13750-024-00343-4)
Supplement: Supplementary file 6 — Additional file 6: Reasons for validity decisions. [file 13750_2024_343_MOESM6_ESM.docx]

README

Title: Reasons for validity decisions

Description: This additional file describes the main reasons for the validity decisions, which were based on the CEECAT tool version 0.2 (Konno, K. 2021. CEECAT: Collaboration for Environmental Evidence Critical Appraisal Tool Version 0.2 (prototype), cited 2021 Jun 2, https://environmentalevidence. org/cee-critical-appraisal- tool/).

Table 1. Main reasons for validity decisions for studies (Article and question (Q) combination) concerning the effects of co-enclosing semi-natural pasture(s) with an improved pasture. Reasons are given in relation to the seven criteria outlined in the CEECAT tool version 0.2 (Konno, K. 2021. CEECAT: Collaboration for Environmental Evidence Critical Appraisal Tool Version 0.2 (prototype), cited 2021 Jun 2, https://environmentalevidence. org/cee-critical-appraisal- tool/). The criteria included in the table are C1: Risk for confounding biases, and C3: Risk of misclassified comparison biases. Reference numbers refer to the reference list in the manuscript reference list. Articles with an (*) contain a study that was included in the quantitative analysis of Q3.

| **Article** | **Q** | **Risk of bias** | **Reason** |
| --- | --- | --- | --- |
| Andrée 2011* [28] | 3 | MEDIUM | Risk for confounding factors, which were not analysed appropriately (C1), no true comparator (C3). |
| Benavides 2009* [55] | 3 | MEDIUM | Risk for confounding factors, which were not analysed appropriately (C1), no true comparator (C3). |
| Celaya 2007* [60] | 3 | MEDIUM | Risk for confounding factors, which were not analysed appropriately (C1), no true comparator (C3). |
| Celaya 2008* [61] | 3 | MEDIUM | Risk for confounding factors, which were not analysed appropriately (C1), no true comparator (C3). |
| Clarke 1995* [58] | 3 | MEDIUM | Risk for confounding factors, which were not analysed appropriately (C1), no true comparator (C3). |
| Ferreira 2012* [62] | 3 | MEDIUM | Risk for confounding factors, which were not analysed appropriately (C1), no true comparator (C3). |
| Ferreira 2013* [63] | 3 | MEDIUM | Risk for confounding factors, which were not analysed appropriately (C1), no true comparator (C3). |
| Ferreira 2017* [56] | 3 | MEDIUM | Risk for confounding factors, which were not analysed appropriately (C1), no true comparator (C3). |
| Hester 1996* [59] | 3 | MEDIUM | Risk for confounding factors, which were not analysed appropriately (C1), no true comparator (C3). |
| Kaufmann 2013* [57] | 3 | MEDIUM | Risk for confounding factors, which were not analysed appropriately (C1), no true comparator (C3). |
| López-López 2015 [64] | 3 | HIGH | C1: Risk for severe confounding factors, which were not controlled for (C1), no true comparator (C3). |
| López-López 2019* [42] | 3 | MEDIUM | Risk for confounding factors, which were not analysed appropriately (C1), no true comparator (C3). |
| Osoro 2005* [65] | 3 | MEDIUM | Risk for confounding factors, which were not analysed appropriately (C1), no true comparator (C3). |
| Pelve 2007 [66] | 3 | MEDIUM | Risk for confounding factors, which were not analysed appropriately (C1), no true comparator (C3). |
| Pelve 2008 [67] | 3 | MEDIUM | Risk for confounding factors, which were not analysed appropriately (C1), no true comparator (C3). |
| Pelve 2010 [68] | 3 | MEDIUM | Risk for confounding factors, which were not analysed appropriately (C1), no true comparator (C3). |
| Pelve 2020 [69] | 3 | MEDIUM | Risk for confounding factors, which were not analysed appropriately (C1), no true comparator (C3). |
| Takala 2015 [24] | 1 | LOW | NA |
| Uytvanck 2010* [29] | 3 | HIGH | Risk for confounding factors, which were not analysed appropriately (C1), no true comparator, not well-defined comparable groups (C3). |

Table 2. Main reasons for validity decisions for studies (Article and question (Q) combination) concerning the effects of supplementary feeding in semi-natural pastures. Reasons are given in relation to the seven criteria outlined in the CEECAT tool version 0.2 (Konno, K. 2021. CEECAT: Collaboration for Environmental Evidence Critical Appraisal Tool Version 0.2 (prototype), cited 2021 Jun 2, https://environmentalevidence. org/cee-critical-appraisal- tool/). The criteria included in the table are C1: Risk for confounding biases, C3: Risk of misclassified comparison biases, and ‘other’, which refers to other reason for increased risk of bias, not identified directly by the criteria. Reference numbers refer to the reference list in the manuscript reference list. Articles with an (*) contain a study that was included in the quantitative analysis of Q3.

| **Article** | **Q** | **Risk of bias** | **Reason** |
| --- | --- | --- | --- |
| Avondo 2002 [70] | 3 | HIGH | No true comparator, unclear effect estimation (C3), supplementary feeding not in focus of study, arbitrary statistics in relation to exposure (other) |
| Bowman 1999* [71] | 3 | MEDIUM | Risk for confounding factors, which were not analysed appropriately (C1). |
| Clariget 2016* [72] | 3 | HIGH | Risk for severe confounding factors, which were not controlled for (C1). |
| Da Ronch 2005 [50] | 1 | HIGH | Risk for severe confounding factors, which were not controlled for (C1). |
| Guerrero 2018* [73] | 3 | HIGH | Risk for severe confounding factors, which were not controlled for appropriately (C1). |
| Mosley 2017* [51] | 1 | MEDIUM | Risk for confounding factors, which were not analysed appropriately (C1). |
|  | 2 | MEDIUM | Risk for confounding factors, which were not analysed appropriately (C1). |
|  | 3 | MEDIUM | Risk for confounding factors, which were not analysed appropriately (C1). |
| Niemelä 2008 [52] | 2 | HIGH | Risk for severe confounding factors, which were not controlled for (C1), not well-defined comparable groups (C3). |
| Ormaechea 2021 [74] | 3 | HIGH | Risk for severe confounding factors, which were not controlled for (C1). |
| Souza 2023 [75] | 3 | HIGH | Risk for severe confounding factors, which were not controlled for (C1). |
| Sowell 2003* [76] | 3 | MEDIUM | Risk for confounding factors, which were not analysed appropriately (C1). |
| Yang 2020 [53] | 2 | MEDIUM | Risk for confounding factors, which were not analysed appropriately (C1). |
|  | 3 | MEDIUM | Risk for confounding factors, which were not analysed appropriately (C1). |
| Yang 2021 [54] | 2 | MEDIUM | Risk for confounding factors, which were not analysed appropriately (C1). |
|  | 3 | MEDIUM | Risk for confounding factors, which were not analysed appropriately (C1). |
