# Supplementary material for: Effects on biodiversity in semi-natural pastures of giving the grazing animals access to additional nutrient sources: a systematic review
Source: Environ Evid. 2024 Aug 1;13:18. doi: 10.1186/s13750-024-00343-4 (PMC11378873; doi:10.1186/s13750-024-00343-4)
Supplement: Supplementary file 11 — Additional file 11: Supplementary figures. [file 13750_2024_343_MOESM11_ESM.docx]

README

Title: Supplementary figures

Description: This additional file contains figures that show 1) the results of the electivity analyses without data from high risk of bias-studies, and 2) the results of the analyses of forage dry matter intake (DMI), with and without data from high risk of bias-studies.


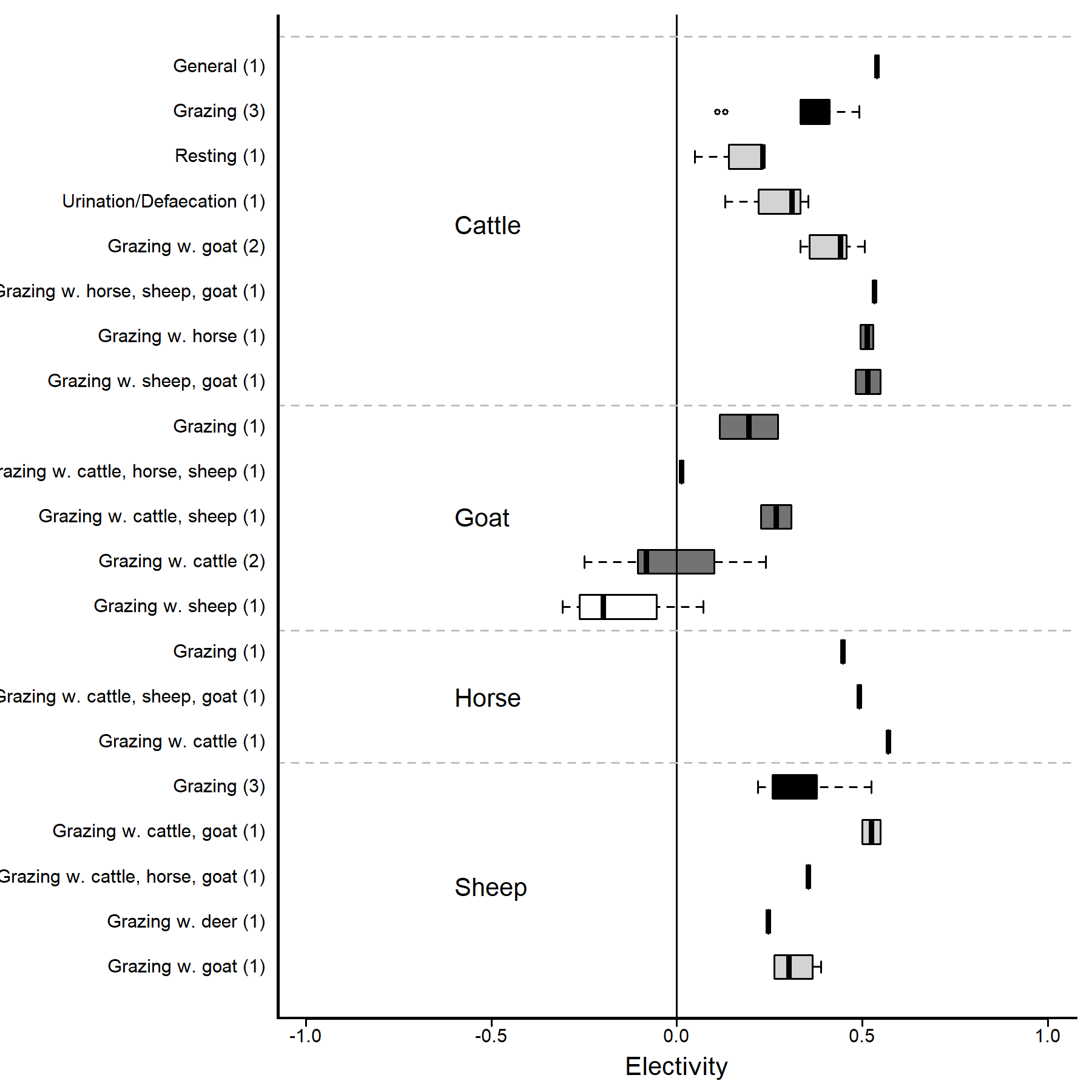


Figure S1. Boxplot of electivity values for improved areas in (semi-)natural pastures for each animal/animal combination with the measured outcome (general, grazing, resting, urination/defaecation) for each year with data from the included studies **except the ones with high risk of bias**. The total number of studies is shown in brackets. Animal combinations are shown as the target species (for which the data relates to) first, and the other species listed after (i.e. if *herd type* = mixed). Electivity values > 0 indicates a preference for the improved area, values < 0 indicates an avoidance of the improved area (values may in theory range from –1 to +1).


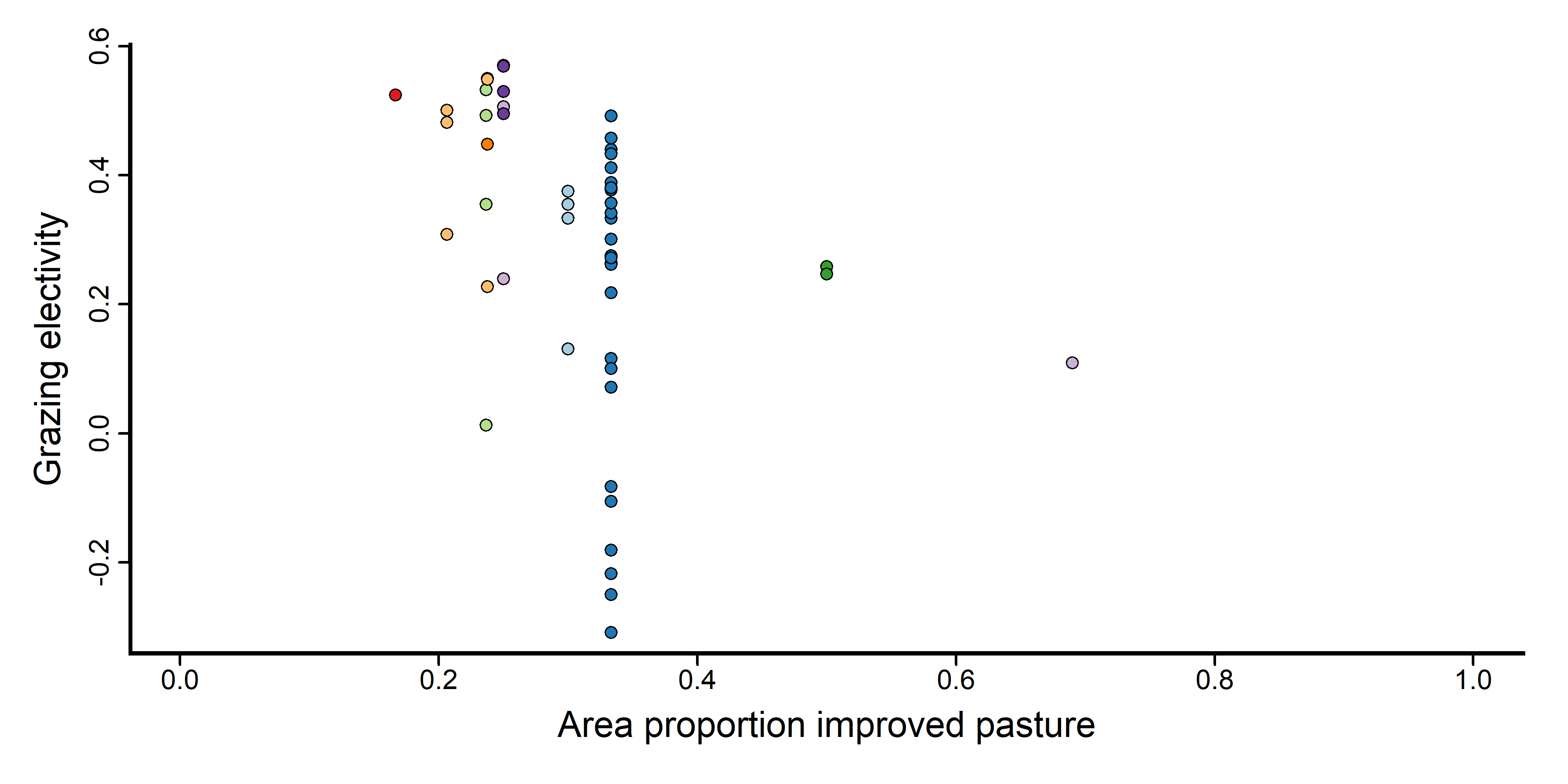


Figure S2. Grazing electivity values for improved areas in (semi-)natural pastures in relation to the *proportion of improved pasture* area within the pasture. Electivity values > 0 indicates a preference for the improved area, values < 0 indicates an avoidance of the improved area (values may in theory range from –1 to +1). The figure shows data from all studies included in the analysis except the ones with high risk of bias (all studies included data on the proportional area of improved pasture), across all *grazer species* and *herd types*.


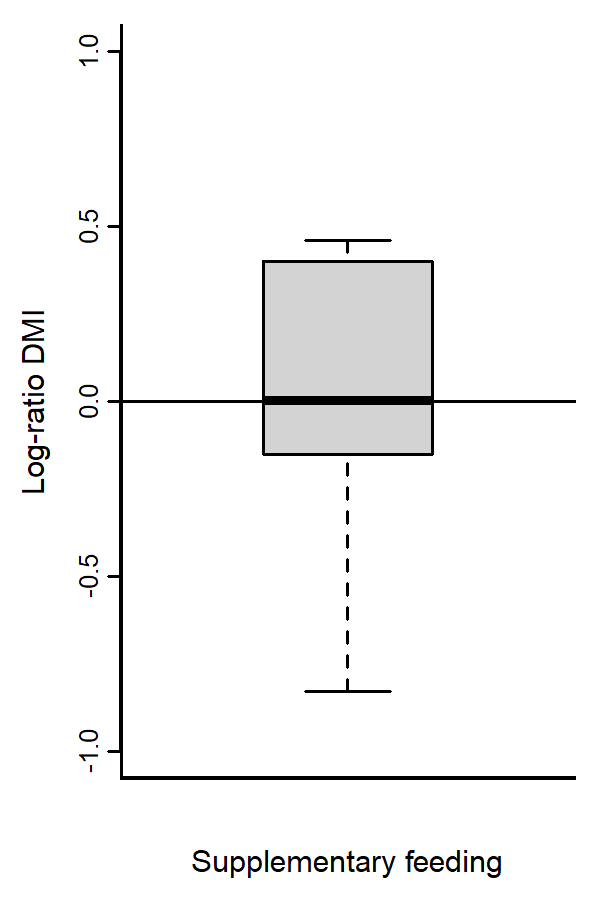

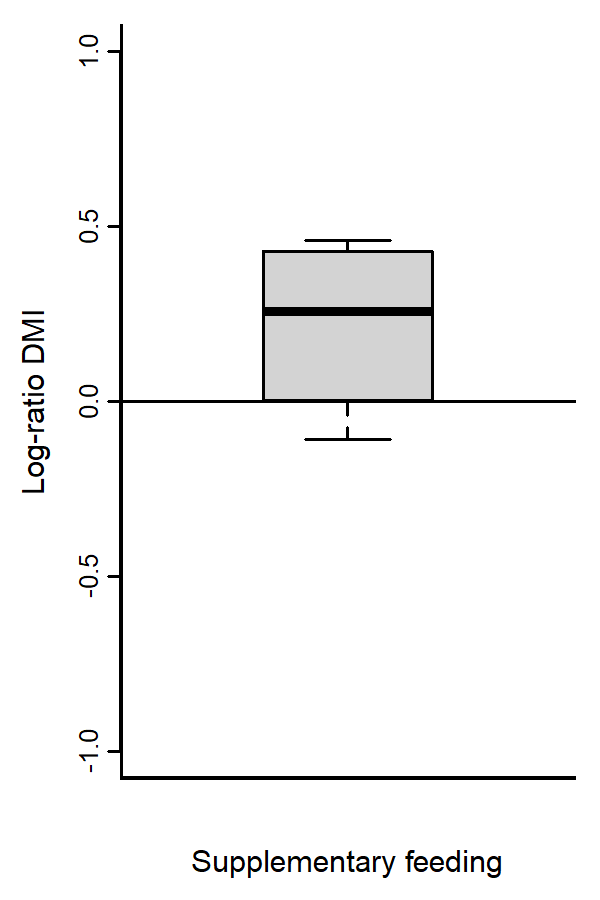


Figure S3a. Boxplot of the log-ratio between forage dry matter intake (DMI) of grazing animals with and without, respectively, supplementary feeding. Log-ratios > 0 indicates a higher intake for animals with supplements, log-ratios < 0 indicates a lower intake for animals with supplements, compared with animals without supplements.

Figure S3b. Boxplot of the log-ratio between forage dry matter intake (DMI) of grazing animals with and without, respectively, supplementary feeding. Log-ratios > 0 indicates a higher intake for animals with supplements, log-ratios < 0 indicates a lower intake for animals with supplements, compared with animals without supplements. The figure **excludes data from studies with high risk of bias**.
